# Enhancing the Cardiovascular Safety of Hemodialysis Care Using Multimodal Provider Education and Patient Activation Interventions: Protocol for a Cluster Randomized Controlled Trial

**DOI:** 10.2196/46187

**Published:** 2023-04-20

**Authors:** Tiffany Christine Veinot, Brenda Gillespie, Marissa Argentina, Jennifer Bragg-Gresham, Dinesh Chatoth, Kelli Collins Damron, Michael Heung, Sarah Krein, Rebecca Wingard, Kai Zheng, Rajiv Saran

**Affiliations:** 1 School of Information University of Michigan Ann Arbor, MI United States; 2 Department of Health Behavior and Health Education School of Public Health University of Michigan Ann Arbor, MI United States; 3 Department of Learning Health Sciences School of Medicine University of Michigan Ann Arbor, MI United States; 4 Department of Biostatistics, Consulting for Statistics, Computing and Analytics Research University of Michigan Ann Arbor, MI United States; 5 National Kidney Foundation New York, NY United States; 6 Division of Nephrology School of Medicine Ann Arbor, MI United States; 7 Kidney Epidemiology and Cost Center University of Michigan Ann Arbor, MI United States; 8 Fresenius Medical Care Waltham, MA United States; 9 Department of Internal Medicine School of Medicine University of Michigan Ann Arbor, MI United States; 10 Veterans Affairs Center for Clinical Management Research US Department of Veterans Affairs Ann Arbor, MI United States; 11 School of Information and Computer Sciences University of California Irvine Irvine, CA United States

**Keywords:** hemodialysis care, patient peer mentoring, telehealth, digital checklist, team training, cluster randomized controlled trial, pragmatic trial

## Abstract

**Background:**

End-stage kidney disease (ESKD) is treated with dialysis or kidney transplantation, with most patients with ESKD receiving in-center hemodialysis treatment. This life-saving treatment can result in cardiovascular and hemodynamic instability, with the most common form being low blood pressure during the dialysis treatment (*intradialytic hypotension* [IDH]). IDH is a complication of hemodialysis that can involve symptoms such as fatigue, nausea, cramping, and loss of consciousness. IDH increases risks of cardiovascular disease and ultimately hospitalizations and mortality. Provider-level and patient-level decisions influence the occurrence of IDH; thus, IDH may be preventable in routine hemodialysis care.

**Objective:**

This study aims to evaluate the independent and comparative effectiveness of 2 interventions—one directed at hemodialysis providers and another for patients—in reducing the rate of IDH at hemodialysis facilities. In addition, the study will assess the effects of interventions on secondary patient-centered clinical outcomes and examine factors associated with a successful implementation of the interventions.

**Methods:**

This study is a pragmatic, cluster randomized trial to be conducted in 20 hemodialysis facilities in the United States. Hemodialysis facilities will be randomized using a 2 × 2 factorial design, such that 5 sites will receive a multimodal provider education intervention, 5 sites will receive a patient activation intervention, 5 sites will receive both interventions, and 5 sites will receive none of the 2 interventions. The multimodal provider education intervention involved theory-informed team training and the use of a digital, tablet-based checklist to heighten attention to patient clinical factors associated with increased IDH risk. The patient activation intervention involves tablet-based, theory-informed patient education and peer mentoring. Patient outcomes will be monitored during a 12-week baseline period, followed by a 24-week intervention period and a 12-week postintervention follow-up period. The primary outcome of the study is the proportion of treatments with IDH, which will be aggregated at the facility level. Secondary outcomes include patient symptoms, fluid adherence, hemodialysis adherence, quality of life, hospitalizations, and mortality.

**Results:**

This study is funded by the Patient-Centered Outcomes Research Institute and approved by the University of Michigan Medical School’s institutional review board. The study began enrolling patients in January 2023. Initial feasibility data will be available in May 2023. Data collection will conclude in November 2024.

**Conclusions:**

The effects of provider and patient education on reducing the proportion of sessions with IDH and improving other patient-centered clinical outcomes will be evaluated, and the findings will be used to inform further improvements in patient care. Improving the stability of hemodialysis sessions is a critical concern for clinicians and patients with ESKD; the interventions targeted to providers and patients are predicted to lead to improvements in patient health and quality of life.

**Trial Registration:**

ClinicalTrials.gov NCT03171545; https://clinicaltrials.gov/ct2/show/NCT03171545

**International Registered Report Identifier (IRRID):**

PRR1-10.2196/46187

## Introduction

### Background

End-stage kidney disease (ESKD) occurs when a person’s kidneys are severely damaged and can no longer function independently. Patients with ESKD require dialysis or transplantation to survive. In 2020, 480,516 Americans (61.3% of patients with ESKD) received outpatient, facility-based hemodialysis (also known as in-center hemodialysis) [1]. This includes 109,107 new patients undergoing hemodialysis in 2020 [1]. Diabetes and hypertension are the leading causes of ESKD in the United States [1]. Accordingly*,* most patients with ESKD have multiple chronic conditions, including cardiovascular disease. In particular, ventricular hypertrophy and varying degrees of heart failure are common among patients on dialysis, compounded by the effect of intermittent fluctuations in blood volume during intermittent (eg, thrice weekly) hemodialysis [2]. The annual mortality rate among patients on dialysis in the United States has increased by nearly 17% during the COVID-19 pandemic in 2020. However, more than half of the deaths were owing to cardiovascular disease [1]. Patients on hemodialysis also experience low quality of life [3] and considerable pain, fatigue, social restrictions, and distress [4-7].

Patients on hemodialysis treated in a dialysis treatment facility typically receive dialysis treatment sessions 3 times a week, for 12 hours per week. On average, 20% of sessions become unstable, most commonly from low blood pressure, or *intradialytic hypotension* (IDH) [8-11]. IDH, a form of cardiovascular and hemodynamic instability, affects half of all patients on hemodialysis [8-11]. IDH may precipitate cramping, dizziness, vomiting, fainting, and fatigue [4,12,13]. The chief causes of IDH are removal of more fluid in a single session than a patient can tolerate, or removal of fluid faster than a patient can tolerate [14]. In the face of preexisting diminished cardiovascular reserves, this often results in hemodialysis instability and repeated myocardial hypoperfusion with resultant cardiac wall motion abnormalities, a phenomenon referred to as *myocardial stunning* [15-17]. Evidence shows that such fluid removal may be responsible for injury resulting from hypoperfusion in other organs, including the central nervous system (eg, repetitive neurological injury leading to cognitive dysfunction) [18].

Cardiovascular and hemodynamic instability during hemodialysis sessions may be preventable. Session stability is determined by the interplay between multiple factors, many of which are modifiable. At the patient level, these factors include decisions regarding sodium and fluid intake and skipping or shortening sessions [19]. Moreover, because early intervention in an IDH episode is important to prevent worsening, patients can notify providers of related symptoms, such as nausea, vomiting, dizziness, or muscle cramping, facilitating prompt provider-based interventions such as placing the patient in the Trendelenburg position [20]. Clinician practice patterns influencing session stability include the physician’s decisions regarding the patient’s target posthemodialysis weight (sometimes called “estimated dry weight”, or “EDW” in practice) and prescribed treatment time and how often these are revisited. Facility-level policy or practice decisions such as a threshold for fluid removal speed (ultrafiltration rate [UFR]) and flexibility toward adding an extra session per week for specific patients are also potentially modifiable factors.

Owing to the relatively recent scientific consensus regarding the cardiovascular harm of rapid fluid removal [21], hemodialysis care providers may not always routinely incorporate cardiovascular and hemodynamic stability into their patient care decisions. In other words, providers regularly choose hemodialysis session lengths, fluid removal targets, and UFRs in ways that affect cardiovascular and hemodynamic stability, perhaps without direct attention to this factor. To provide a better foundation for such decisions, a consensus statement from Medical Directors of United States hemodialysis facilities called for trials of methods for improving patient fluid management [22], we address this call in our research.

### Objectives

We will independently evaluate and compare the effects of 2 facility-level interventions (multimodal provider education and patient activation interventions) on the cardiovascular and hemodynamic stability of hemodialysis care. Briefly, the multimodal provider education intervention includes a tablet-based checklist and team training for dialysis facility staff. The patient activation intervention includes tablet-based educational modules and peer mentoring. We pursue our study objectives through the following specific aims:

1. Aim 1: To conduct a cluster randomized controlled clinical trial to test and compare the effects of 2 hemodialysis facility-level interventions on the primary outcome of hemodialysis session stability over an intervention period of 24 weeks and a postintervention follow-up period of 12 weeks.
2. Aim 2: To test and compare the effects of 2 hemodialysis facility-level interventions on secondary patient-centered clinical outcomes, including patient symptoms, fluid adherence, hemodialysis adherence, quality of life, hospitalizations, and mortality over the same time frame.
3. Aim 3: To identify factors associated with the successful implementation of the interventions and ways in which implementation may influence intervention effectiveness.

### Hypotheses

Our main study hypothesis is that hemodialysis session stability will significantly improve with either multimodal provider education or patient activation interventions, and that multimodal provider education will show a greater magnitude of improvement. This hypothesis is based on our expectation that some patients on hemodialysis might refuse or be unable to participate in the peer mentoring component of the patient activation intervention, potentially leading to greater reach of the provider intervention. We will test this main hypothesis and explore this potential explanation of any differential improvement as part of our planned sensitivity, mediation, and moderation analyses.

## Methods

### Study Design

We have translated 2 evidence-based interventions from their previous applications in inpatient safety (checklists [23] and team training [24]) and chronic disease self-management interventions (peer education and mentoring [25,26]) into the outpatient hemodialysis care context [27-29]. We will conduct a cluster randomized controlled clinical trial in 20 dialysis facilities to independently test and compare the effectiveness of each of the 2 interventions for improving the primary outcome of hemodialysis session stability, measured as the occurrence of IDH during a given session. The cluster randomized controlled trial design [30] is most appropriate to our goal of improving routine hemodialysis care. Because pragmatic intervention studies are most suited to supporting health care-related decision-making [31], the effectiveness of the interventions will be assessed under the usual conditions in which they will be applied [31]. The study will also use outcome data already gathered routinely by hemodialysis facilities [32].

Facilities will be randomized in a 2 × 2 factorial design. We selected this design because it is statistically efficient in providing a control group for testing each intervention. Furthermore, this design facilitates the comparison of the interventions’ relative magnitudes of the effects and their potentially synergistic or antagonistic effects.

For aim 1 activities, the primary outcome, IDH occurrence, will be monitored for 12 weeks before the intervention (baseline period) for 24 weeks during the intervention period and for 12 weeks after the intervention period (follow-up period). We will also assess the effects of the interventions on secondary outcomes, including patient symptoms, fluid adherence, hemodialysis adherence, quality of life, hospitalization, and mortality (aim 2). The implementation of the interventions will also be assessed (aim 3).

### Ethics Approval

This study has obtained institutional review board (IRB) approval for a waiver of informed consent and waiver of Health Insurance Portability and Accountability Act (HIPAA) authorization based on the “no more than minimal risk with potential direct benefits” to patients and the study’s pragmatic nature (HUM00125305).

### Study Organization and Partners

This study is led by a research team at the School of Information, Medical School, and the School of Public Health at the University of Michigan, Ann Arbor, United States. The study’s data coordinating center, based in the Kidney Epidemiology and Cost Center at the School of Public Health, will receive deidentified patient data from a large dialysis provider organization to which all facilities in the study will belong. The National Kidney Foundation (NKF), a partner in the study, created patient educational module content and will recruit and train peer mentors for facilities assigned to the patient activation study arms. The patient activation intervention application used by peer mentors and their patient mentees will be developed by collaborators at the University of California, Irvine and at a telehealth platform company, VSee Inc. Funding for this study has been provided by the Patient-Centered Outcomes Research Institute.

### Eligibility and Recruitment of Facilities

A total of 20 hemodialysis facilities were recruited from the following regions in the United States: Midwest, Northeast, Southeast, and Southwest. Facility inclusion criteria were as follows: (1) outpatient hemodialysis facilities, (2) at least 70 adult patients (aged ≥21 years) who were permanent patients at the study facility, (3) not currently involved in another study, and (4) no operational or administrative reasons that would make the trial difficult to implement at that site. Hemodialysis facility staff and providers will not be participants in the research (ie, data will not be collected from or about individual staff to answer the study research questions). Facilities were stratified by county poverty rate and then randomly selected to prevent one region or arm of the study from having a different socioeconomic status profile than the others.

### Eligibility and Recruitment of Patients

As this is a pragmatic trial, we aim to include all nonvulnerable (see definition of vulnerable below in this section) in-center patients at participating hemodialysis facilities. The trial was designed so as not to exclude any patient, including women and minorities to the extent they are represented at the hemodialysis facilities from the age group of ≥21 years. We will only exclude vulnerable patients, including those below the age of 21 years; prisoners; those with a cognitive barrier; those deemed vulnerable by the clinical manager, medical director, or social worker; and those who are unable to comprehend the patient information sheet because of lack of facility in reading English or Spanish. Following prior pragmatic research in hemodialysis care [33] and the study’s IRB review results, the study uses an “opt out” process for using patient clinical data to determine test facility-level and patient-level outcomes. Each patient will have the opportunity to opt out of having their data used in the study for outcome monitoring as part of aim 1 and 2 activities. The University of Michigan will manage the opt-out process and provision of study information remotely with the assistance of facility staff. Each patient will be given an information sheet by dialysis facility staff; these staff will also be provided an IRB-approved script to use when providing patients with the information sheet. Facility staff will record that each patient has received the information sheet and if they have verbalized a decision to opt out of the study. The information sheet will also provide a 1-800 telephone number to call the University of Michigan research team to learn more about the study or opt out of the study at any time if they desire. Patients may opt out of the study at any time, but patients in facilities assigned to the provider intervention arms of the study will still benefit from multimodal provider education as this is a facility-level intervention that does not involve specific patients.

For the patient activation intervention only, we will randomly select patients, in succession, who are not vulnerable or have not opted out of the overall study, until the target number of patients in a facility accept peer mentoring. That is, every time a patient declines to participate, we will choose another patient randomly until the enrollment target is reached. On the basis of prior studies [34-36], we estimated a retention rate of at least 73% for the peer mentoring intervention.

### Detailed Intervention Description

#### Multimodal Provider Education Intervention

**Figure 1:**
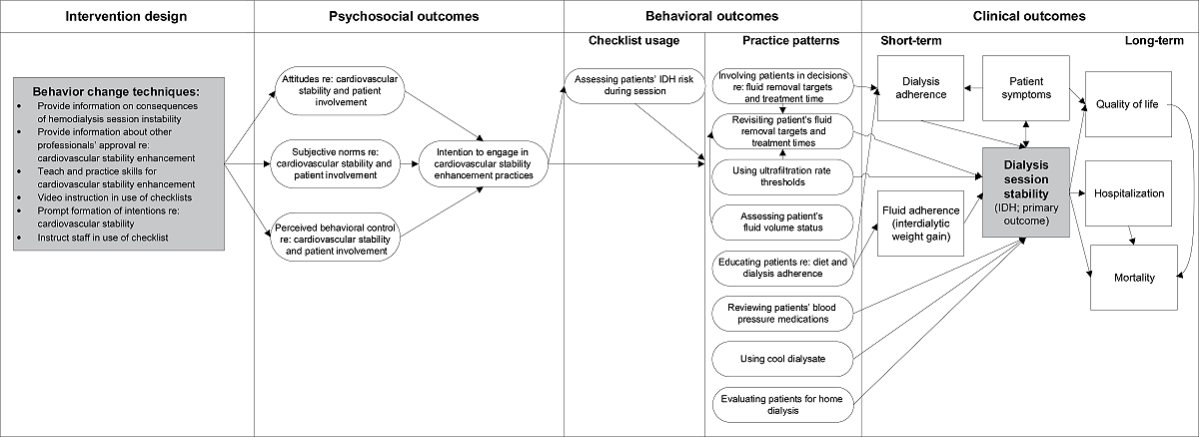
Theory-based model of behavior change for multimodal provider education intervention. IDH: intradialytic hypotension.

As Figure 1 shows, the provider intervention is informed by the Theory of Planned Behavior, commonly used in practice change interventions involving clinicians [37,38], including a study assessing the adoption of a patient safety checklist [39]. Using this model, we predict that changes in perceived behavioral control and behavioral intentions [40,41] resulting from the training will change providers’ fluid management practice patterns (including use of the checklist), which will in turn reduce rates of hemodialysis session instability.

There are 2 components to the provider intervention. The first component consists of 4 training modules that will be completed by nurses, patient care technicians, dieticians, and social workers in the study facilities. The goals of these sessions were as follows: (1) to educate facility staff on the risks of IDH, (2) to promote recognition of IDH risk factors and opportunities for prevention and early intervention, and (3) to instruct staff in use of the checklist. Staff training will be implemented by the project coordinators at University of Michigan. It will include 2 self-paced modules available on web through the facility’s access to a web-based learning management system and 2 synchronous web-based meetings between the project coordinators and staff at the facilities randomized to receive the provider intervention (Textbox 1 provides an overview of the staff training sessions).

In addition, physicians, nurse practitioners, and physician assistants who are not part of the hemodialysis facility staff, but who round in study facilities, will have access to a 1-hour asynchronous web-based training module. This training program reviews the purpose of the study and its scientific rationale. It also prepares them for potential treatment changes that facility staff, particularly nurses, may discuss with the provider as a result of using the checklist.

**Figure 2:**
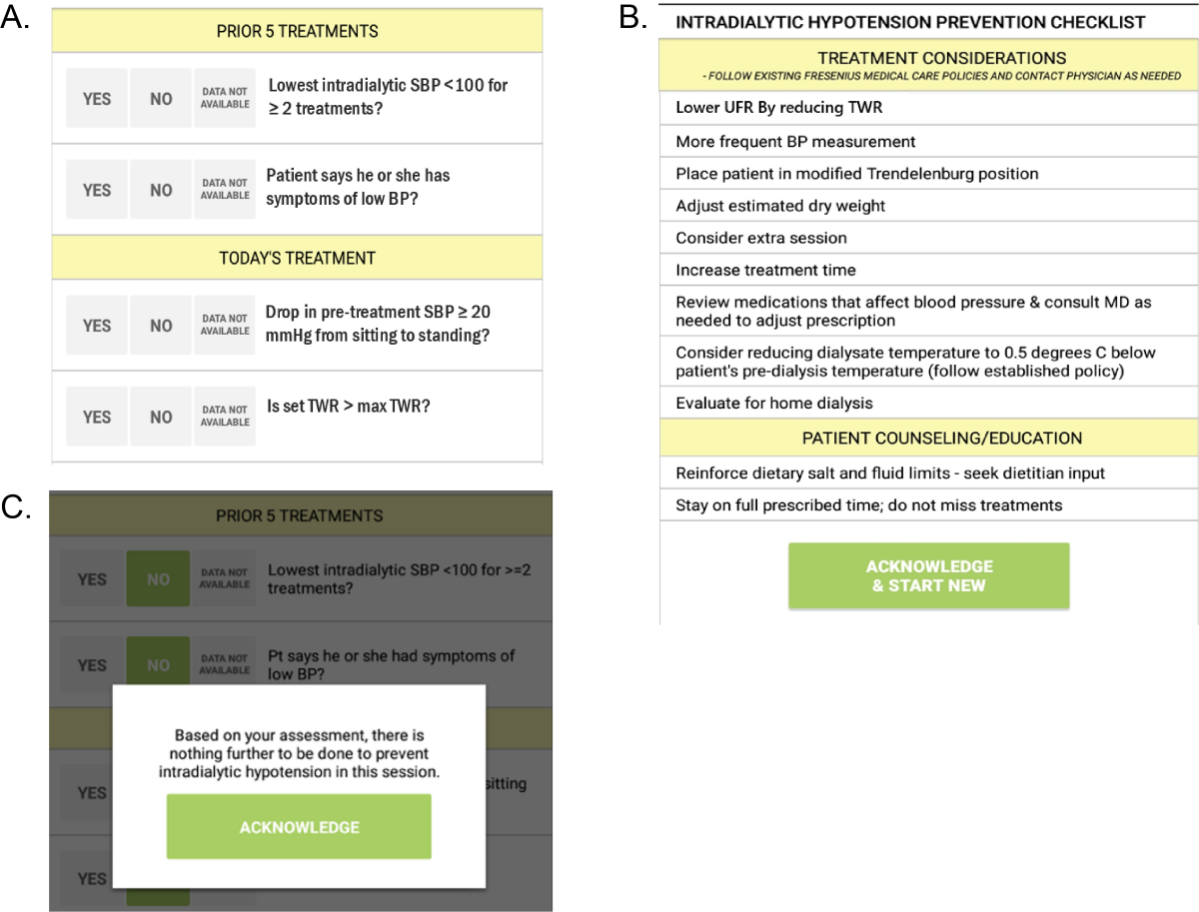
Intradialytic hypotension (IDH) prevention checklist. A) Questions asked at each dialysis session to all patients in clinics assigned to the provider intervention. B) Suggested actions for dialysis care team if any questions from the checklist (A) are answered “yes” or “data not available.” C) Screen displayed if ALL questions from the checklist (A) are answered “no ”. BP: blood pressure; SBP: systolic blood pressure; TWR: target weight removal; UFR: ultrafiltration rate.

The second intervention component is the IDH prevention checklist [27], which will be provided on tablet computers (Figure 2). The checklist aims to identify patients at increased risk of IDH; it is to be completed by a nurse for each patient’s hemodialysis treatment with verbal input from the patient and patient care technician as indicated. The checklist has 4 items that may be associated with increased IDH risk: 2 pertaining to the past 5 treatments and 2 pertaining to the current session. If any risk factors are identified, a list of suggestions based on the evidence-based literature on how to prevent IDH will be presented. Suggestions include measures for preventing IDH in the current hemodialysis session (ie, placing the patient in a modified Trendelenburg position) as well as longer-term changes that may require a physician’s input (ie, adjusting the patient’s estimated dry weight or providing additional counseling on sodium restrictions). The data collected through the checklist will not identify the patient or the staff member, but the use will be automatically logged on a HIPAA-compliant server, including the facility, date, time, items displayed, and items clicked.

**Overview of the staff training intervention.**

- The first training is a self-paced module through the learning management system available to the facilities. It will introduce the Dialysafe project goals and study design. Staff will refresh their knowledge on consequences of intradialytic hypotension (IDH) and identify the training requirements of their assigned study group.
- The second training is designed for active participation and discussion from the providers. Staff will consider the most susceptible points in time for patients to experience cardiovascular instability, including IDH. The training will review principles of weight and blood pressure measurement per existing facility policy. The audience will be introduced to how the nurse will use the Dialysafe IDH prevention checklist to systematically evaluate every patient for IDH risk early in the session, and how to make changes to the hemodialysis session if the patient is found to be at risk.
- In the third training, staff will review actions for increasing cardiovascular and hemodynamic stability for patients at higher risk of IDH. Key points of this training include the importance of frequently assessing a patient’s dry weight and making changes as necessary, the need to review blood pressure medications, and the use of cool dialysate. These topics are some of the methods that can be used to prevent IDH; providers will also be given time to reflect on and discuss other measures they have found effective. Finally, staff will review their role in IDH prevention and consider times when they might discuss making changes to a patient’s hemodialysis sessions. They will also reflect on their experience with the Dialysafe IDH prevention checklist.
- Staff training part 4 is a 15-question multiple-choice quiz hosted on the learning management system.

#### Approaches to Encouraging Participation and Retention for Provider Intervention

Several implementation strategies will be used to promote the successful adoption of the provider intervention. All facilities randomized to this intervention will appoint a *study champion* who will help convey positive messages about the study, including staff training and checklist implementation. Each of these facilities will also form an operations committee, which will include the study champion, an area manager, patients, and others selected to represent different organizational levels and perspectives, as intervention implementation will be enhanced by the early involvement of staff at various levels [42].

Checklists are more likely to be effectively used if accompanied by training that addresses the *why* and *how* questions regarding their implementation [43-46]. Therefore, the second provider training includes how to use the checklist and its associated rationale. The third training includes dedicated time for discussing the ways in which the checklist has or has not been integrated into the facility workflow to promote the sharing of learning and process improvements [47].

Notably, both components of the multimodal provider education intervention offer incentives for completion. We plan to offer incentives to staff in facilities that consistently use the checklist. For each month of intervention, facilities that achieve a 90% checklist completion rate will be provided lunch. Facilities that achieve a 90% checklist completion rate for the entire study will receive a certificate of recognition that can be posted in an area visible to staff and patients.

Facility-level incentives include the fact that study facilities that participate in trainings 2 and 3 and meet the requirements will receive one “Diamond” toward its 5-Diamond status. The 5-Diamond Patient Safety Program is a national initiative. The 5-Diamond Patient Safety Program was designed to assist dialysis centers with safety standards through continuous learning modules for frontline clinicians. Participating dialysis facilities are required to complete a series of safety modules each year to earn or renew their 5-diamond status, and annual participation in the 5-Diamond Patient Safety Program is already undertaken at all hemodialysis facilities in this study. In addition to the facility’s diamond status, nurses and patient care technicians who attend these training sessions will be offered optional continuing education unit credit. Physicians and advanced practitioners will also be able to earn continuing medical education or continuing nursing education credit by watching the 1-hour training video described previously. Therefore, successful completion of the provider training offers both facility-level and individual-level incentives.

#### Patient Activation Intervention

**Figure 3:**
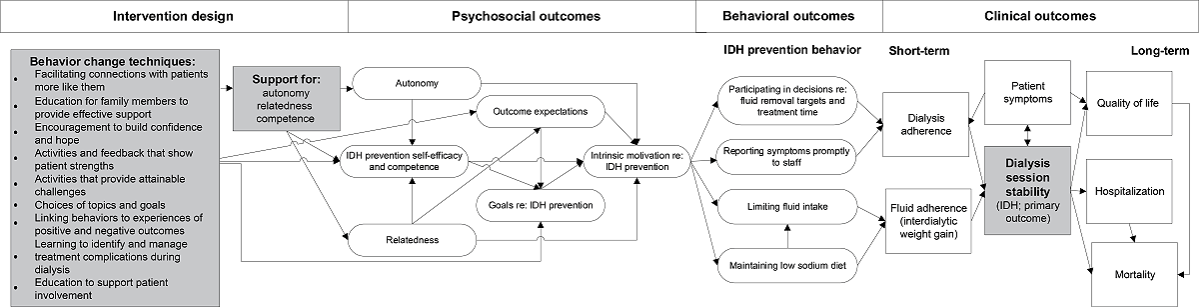
Theory-based model of behavior change for patient intervention. IDH: intradialytic hypotension.

The patient activation intervention includes both internet-based educational modules and peer mentoring. Both intervention components were designed using Social Cognitive Theory and Self-determination Theory [28]. Social Cognitive Theory [48-50], an approach with strong empirical support in evidence-based interventions, is a key framework underlying the patient intervention. Social Cognitive Theory emphasizes the predictive power of *self-efficacy*, or confidence in one’s ability to perform an action, in the likelihood of performing that action (Figure 3). This theory has led to successful peer-based chronic disease interventions [51,52]. The design of the educational modules is also informed by Self-determination Theory [53], which proposes that people are more likely to engage in a behavior when they are intrinsically motivated to perform it. Intrinsic motivation emerges when one finds an activity enjoyable or satisfying, as is the case when one’s psychological needs are met when performing that activity. Self-determination Theory highlights three psychological needs: (1) autonomy (the need to feel in control of one’s life), (2) competence (the need to feel effective in dealing with situations one encounters), and (3) relatedness (having meaningful relationships or having a sense of belonging in a community).

The patient activation intervention will be digitally available on tablets shipped to patients for use at home and shipped back to the university upon intervention completion. The patient activation intervention is delivered through 5 modules to be completed roughly 1 week apart from one another. Each module focuses on a different aspect of preventing IDH (Table 1 provides a detailed summary of the module’s content and related behavioral goals). Each module includes the following elements: (1) a slide deck presented as an informational video; (2) an optional quiz about the video content; (3) patient experience stories presented as short video clips; (4) a goal-setting feature; (5) the option to view or email supplemental NKF handouts and suggested apps; (6) a peer mentoring session lasting 20 minutes to 1 hour supported by a discussion guide for mentors; and (7) an action plan with patient-identified goals, values, and steps to overcome barriers. The action plans will be completed by the peer mentor and emailed to the patient after the mentoring session. Multimedia Appendices 1 and 2 provide screenshots of the digital modules.

Peer mentoring sessions will be delivered via a secure, HIPAA-compliant videoconferencing platform. The intervention extends an existing peer mentoring program offered by NKF. NKF Peer Mentors are trained patients with ESKD who volunteer to support other patients. Mentors can speak from their own experience about the challenges of life on hemodialysis; thus, patients often view mentors as relatable, credible, and accessible information sources [51,54-56]. Mentors can serve as role models to encourage positive health behavior change as they share their experiences [55]. In this study, mentors will receive additional training on study-specific information and motivational interviewing. Accordingly, during the sessions, peer mentors will use discussion guides that implement motivational interviewing [57] principles based on Self-determination Theory [53]. Motivational interviewing involves encouragement, exploration of feelings, and personalized goal setting. It has been effective across clinical contexts [58-63] including for behavior change [64,65].

NKF is recruiting, training, and supporting mentors for involvement in the study. The mentors will receive study-specific training in addition to standard peer mentor training offered by NKF. Peer mentor training will be divided into 10 self-paced training modules, with one live, internet-based training session for role plays and mentor skill assessment. Training will include information on: (1) the role of the peer mentor; (2) hemodynamic stability; (3) listening and communication skills, including motivational interviewing techniques; (4) sharing experiences and strategies; (5) confidentiality and boundaries; and (6) program logistics such as using multimedia tools, intervention fidelity, and record keeping. After mentors have completed all the training modules, they will be matched with ≥1 mentees who have consented to participate in the program as part of the intervention at their facility.

**Table 1:** Interactive education modules for patient intervention.

Topic	Content	Behavioral goals
Getting enough dialysis	How dialysis makes patients feel better Importance of removing fluid and preventing fluid overload	Attending dialysis sessions for the full length of time Understanding benefits of attending lengthened or supplemental dialysis sessions as needed Identifying values that will help with goal setting
Feeling better with less salt or sodium	Why people on dialysis need a low-sodium diet How to read a food label Examples of low and high sodium foods	Maintaining a diet that is low in sodium Tracking sodium intake
Making fluid restrictions work for you	Why people on dialysis need to limit fluid intake Ways to decrease thirst and track fluid intake	Maintaining a level of fluid intake that will optimize interdialytic weight gain Becoming aware of usual interdialytic weight gain Tracking fluid intake Discussing symptoms of fluid overload with the dialysis team Discussing the need for longer or extra sessions if experiencing fluid overload symptoms
Feeling better on dialysis and having easier sessions	Strategies to prevent low blood pressure and other symptoms of fluid removal Steps to correct low blood pressure Why it is helpful to check blood pressure at home	Recognizing symptoms of IDH^a^ Reporting IDH symptoms and episodes to staff Tracking blood pressure at home Notifying staff of changes in true body weight
Getting more involved in your care	Patient roles and responsibilities as a member of the health care team Roles and responsibilities of other dialysis team members	Actively participating in decisions with providers regarding fluid removal targets, ultrafiltration rates, and treatment times Asking questions of the dialysis team relevant to session stability Share concerns related to health and care, emotional well-being, and upcoming procedures with the dialysis team

^a^IDH: intradialytic hypotension.

#### Approaches to Encouraging Participation and Retention for Patient Intervention

Although participant retention is an issue in virtually any trial, patients on hemodialysis are likely to experience fatigue, pain, and other symptoms; thus, there is a greater than average risk of patients not completing the intervention. Therefore, we will offer incentives [61] for both mentors and mentees to encourage completion of all 5 peer mentoring sessions. Mentors will receive US $100 per patient with whom they work and US $15 for completion of the peer mentoring satisfaction survey. Mentees will receive a total of US $120 for completing the entire mentoring program and peer mentoring satisfaction survey. Although mentors will be paid upon completion of the mentoring sessions, mentees will be paid on a per-session basis at an escalating rate (US $10 for session 1, US $15 for session 2, US $20 for session 3, US $25 for session 4, and US $50 for session 5 and completion of the satisfaction survey).

In addition, we will use several general evidence-based retention strategies: issuing reminders before a scheduled mentoring session [66], monitoring satisfaction with the study [67] via the planned peer mentoring survey (Multimedia Appendix 3 provides the survey instruments), and keeping the response burden for patient data collection very low [68].

### Data Collection

For aim 1 and 2 activities, most of the data used in this study are already routinely documented within the electronic health record of participating hemodialysis facilities. Additional information gathered during this study will be minimal because of the study’s pragmatic nature.

#### Primary Outcome

Our primary outcome is hemodialysis session stability, specifically IDH, which is measured every session using blood pressure cuffs worn by patients. The primary indicator of IDH, based on direct blood pressure measurements after the start of the session, will be sitting systolic blood pressure (SBP) falling below 100 mm Hg (using the lowest SBP during the session) if starting SBP ≥100 mm Hg.

Secondary measures of IDH based on direct blood pressure measures will include the lowest (minimum) valid SBP below 100 mm Hg during the session if starting SBP is ≥100 mm Hg; the number of SBP measurements <100 mm Hg (using raw blood pressure measures, time of blood pressure measurement, and starting SBP) if the starting SBP is ≥100 mm Hg; and whether the sitting SBP falls below 90 mm Hg (using the lowest SBP during session) if starting SBP ≥100 mm Hg.

#### Justification of Selection of Primary Outcome

IDH is linked to symptoms such as cramping, dizziness, vomiting, fainting, and fatigue [4,12,13,69] and increased risk of other outcomes important to patients including cardiovascular disease [70], hospitalization [71], and mortality [72-74]. IDH is a top-10 priority outcome (of 33 ranked) for patients on hemodialysis [75]. Patients express concern about the receipt of insufficient hemodialysis or administration of fluids in cases of IDH [69].

There is a lack of consensus on IDH definitions; however, for the purposes of this study, we use the threshold of SBP <100 mm Hg. As documented in the literature, a drop in SBP to <100 mm Hg is associated with a higher probability of patient-reported negative symptoms [76]. Even in asymptomatic events, intervention at this threshold has the potential to prevent repetitive multi-organ tissue ischemia [77] and further decline to dangerously low levels of SBP <90 mm Hg [71,72]. A lower threshold (eg, SBP <90 mm Hg or the occurrence of symptoms or clinical interventions) would not be appropriate for this study as we aim to prevent organ damage and negative patient experiences [5].

#### Secondary Outcomes

The patient-centered secondary outcomes are described in the following subsections.

##### Interdialytic Weight Gain

Facilities record this measure of fluid gain between hemodialysis sessions by measuring the difference between patients’ pre- and posthemodialysis weights at each hemodialysis session. This outcome is important to patients as high fluid gains are linked to symptoms such as bloating and shortness of breath [78]. High fluid gains are associated with fluid overload, which is a leading cause of hospitalizations in patients on hemodialysis [79,80]. High fluid gains are also associated with mortality risk, particularly from cardiovascular causes [81,82]. Interdialytic weight gain is a top-20 priority outcome (of 33 ranked) for patients on hemodialysis [75].

##### Hemodialysis Adherence

Facilities routinely collect data on hemodialysis adherence [83]. We will use 3 measures: number of minutes of prescribed hemodialysis time missed per week, number of missed sessions per week, and total missed session time.

##### Patient Symptoms

Symptoms during the hemodialysis session are a priority outcome for patients [84]. Symptoms such as severe fatigue and cramping are reported in 50% and 30% of hemodialysis sessions, respectively [4]. We will create a patient symptom burden measure combining symptom frequency and severity for each of the following symptoms that are often indicative of IDH, if they occur during or after the hemodialysis session (not prehemodialysis): nausea, vomiting, abdominal pain, dizziness, muscle cramps, headache, chest pain, shortness of breath, palpitations, diaphoresis, and blurred vision. Patients may report symptoms to the dialysis team or nurses to assess symptoms at each hemodialysis session and document them at that time. We will also add the symptoms listed on the Kidney Disease Quality of Life survey to the patient symptom burden measure. We will also calculate the proportion of sessions with each symptom using date and time of session.

More than 40% of patients feel that they have not fully recovered even after returning home after hemodialysis [4]. As an indicator of both fatigue and energy and hemodialysis-free time, this is one of the most important outcomes to patients on hemodialysis (first and fourth of 33 ranked) and more important to patients than to health care providers [75,85]. Therefore, two additional questions will be answered in the electronic health record documentation during the midweek hemodialysis session for all patients in the study:

1. After your last dialysis session, how long did it take you to recover enough to do the things you normally do on a nondialysis day?
2. Did the patient make a request today regarding their fluid removal goal?

The first question was created in consultation with the dialysis provider’s patient advisory committee and our study’s patient partners and builds on past research [86]. Responses to this question will also be used as a secondary outcome for patient symptoms alone and in combination with the patient symptom burden measure described earlier.

##### Health-Related Quality of Life

This important patient-reported outcome [87] will be measured before and after the intervention. Health-related quality of life is a longer-term measure that reflects symptom patterns and other key life dimensions, including physical functioning, emotional well-being, pain, and energy levels [88]. The suffering represented by low health-related quality of life predicts outcomes that matter to patients, including hospitalization [89] and mortality [89,90].

We will use the 36-item Kidney Disease Quality of Life (KDQOL-36 version 1) [91,92], which has good internal consistency based on α scores [92]. This instrument is required annually for all patients on hemodialysis in the United States [93]. The KDQOL results will be provided twice for each patient from the following surveys: (1) the latest survey within 12 months before the start of the study intervention and (2) the earliest survey within 12 months after the start of the intervention.

##### Hospitalizations

We will gather these data from the facility’s records for all patients within the study period. Hospitalizations represent a period of heightened symptoms and lost functioning among patients; patients describe hospitalizations as extremely distressing, and they are a top-21 priority outcome for patients on hemodialysis (of 33 ranked) [75].

Secondary analyses of these data will assess hospitalizations by cause using hospital discharge diagnosis (eg, fluid-related or cardiovascular causes, COVID-19), number of hospitalizations per patient, and length of hospitalization.

##### Mortality

This has been provided as a key example of a patient-centered outcome [87] and will be gathered from the facility’s patient records.

### Aims 1 and 2: Supplementary Data Collection

#### Practice Patterns Survey

In keeping with the precedent set by the International Dialysis Outcomes and Practice Patterns Study [94], in which several of our team members were involved, we will use practice patterns [94] surveys (Multimedia Appendix 4). These will be delivered at all study facilities during the facility preparation period before the intervention starts and again at the end of the 24-week intervention period. These surveys will also serve as manipulation checks [95] that will allow us to determine whether practice changes occurred at intervention sites while not occurring at the no intervention sites. Accordingly, the survey results will allow us to assess whether the interventions worked as intended. Surveys will be provided to both the nurse manager and the medical director.

#### Peer Mentoring Satisfaction Survey

After the 5 mentoring sessions, both mentees and mentors will receive a satisfaction survey (Multimedia Appendix 3). The results will be analyzed to identify mentees’ experiences with the multimedia educational modules and peer mentoring and the mentors’ experiences with the digital tools, training, and support. We will share the aggregate results with NKF so that they can make program adjustments if needed.

### Aim 3: Data Collection for Assessment of Intervention Implementation

Aim 3 activities will seek to identify variation in implementation approaches adopted within different study facilities and to characterize differences in intervention acceptance and workflow integration. These analyses will help with understanding any potential differences in intervention effects between sites and with identifying and responding to any emergent implementation challenges throughout the trial. We will collect implementation data in all intervention facilities. Data collection activities will not require additional meetings; study staff at University of Michigan will gather data from provider intervention training parts 2 and 3 (10 facilities) and operations committee meetings (15 facilities). No audio or video recordings will be made of any of the meetings or sessions.

At the provider intervention training sessions, UM study staff will take descriptive field notes. The study staff will inform attendees that they will take notes, the purpose of the notes, and that comments made during meetings may be tied to a staff member’s role; however, staff names will not be recorded. The field notes will document the following:

1. Staff questions during the sessions.
2. Staff members’ stated reactions to the interventions.
3. Discussions in which the staff talk about their plans for, and experience with, integrating the provider intervention into their workflows.

To assess usability of the checklist and patient intervention technologies, we will analyze use data, with attention to compiling the following metrics: (1) time spent on each page, (2) number of abandoned checklist sessions and videoconferencing or patient education module sessions, and (3) pages and click-through patterns preceding abandonment. We will present and discuss these data in operations committee meetings. We will also record any staff comments and concerns about the checklist.

The operations committee meeting agendas will also include a small number of questions directly related to study intervention implementation (Multimedia Appendix 5). UM study staff will record answers in descriptive field notes. These questions will be for facility staff, and study staff will ask them after patient representatives have left the meeting.

### Data Analysis

#### Statistical Power

We calculated the statistical power for the study for the primary outcome of hemodialysis session stability. We assumed that a minimum of 60 patients on hemodialysis will be observed for at least 60 hemodialysis sessions and the primary outcome will be the proportion of unstable sessions, which we treat as a continuous variable. We assume that these proportions among patients in a facility are normally distributed with mean 0.20 in the control group and mean between 0.16 and 0.17 in the intervention group (a difference *d* of 0.034-0.043), with SD (or *sigma*) assumed to be in the range of 0.08-0.10. We conservatively assume 60 patients per facility (ie, a total of 60 × 20 facilities=1200 patients). The power calculation is robust to changes in number of patients (m), as the calculation of K (number of clusters) only increases by ~1 if m is increased to 90 [96]. We assume the intraclass correlation coefficient for hemodialysis facilities to be 0.10. We also assumed a significance level of 0.05 in all calculations. If the SD is as large as 0.10, we can detect a difference of 0.043 (ie, 20% vs 16%) with 81% power. In other words, if the smallest variance estimate is correct (sigma=0.08), we have 91% power to detect a 4% difference and 80% power to detect a 3% difference (Table 2).

**Table 2:** Power to detect difference in intradialytic hypotension (IDH) rates. The power is shown to detect a difference in IDH rates (D) between groups with at least (k) number of facilities, assuming 2 possible SDs (sigma).

Detectable IDH rate difference, D	SD, sigma	Number of facilities, k	Power, %
0.034	0.08	10	80
0.034	0.10	10	61
0.040	0.08	10	91
0.043	0.10	10	81

#### Overall Analytical Approach

Our 2 × 2 factorial trial is designed to answer the following four treatment comparison questions (Table 3):

1. Provider education versus no provider education (assumes no interaction between the interventions)

Cells ([A+B] vs [C+D])–*F* test comparison

2. Peer mentoring versus no peer mentoring (assumes no interaction between interventions)

Cells ([A+C] vs [B+D])–*F* test comparison

Note that all data will be reused in the comparisons for 1 and 2. This shows a key advantage of the 2 × 2 design: it allows testing 2 interventions for the “price of one.” Furthermore, if an interaction is observed (question 4 below), then the effect of one intervention can be estimated at each level of the other intervention.

3. Comparative effectiveness of provider education alone versus peer mentoring alone

B versus C–*F* test comparison. The head-to-head comparison of the magnitude of effect for the 2 interventions is actually comparing only the interventions alone, in the absence of the other ones.

4. Are there synergistic or antagonistic effects between the provider education and peer mentoring interventions?

The purpose of this test is to determine whether the effect of one intervention differs depending on the presence of the other intervention. The test compares:

- Cells ([A−C]−(B−D)]–*F* test comparison to examine the effects of provider education intervention with and without the presence of the patient activation intervention.
- This is equivalent to ([A−B]−[C−D]), which examines the patient activation intervention with or without provider education.

**Table 3:** A 2 × 2 factorial trial design with facility and patient sample sizes for treatment comparisons.

			Patient activation intervention, number of facilities (number of patients)					Total, number of facilities (number of patients)
			Yes		No			
**Provider education**								
	Yes	A—5 (300)		B—5 (300)		10 (600)		
	No	C—5 (300)		D—5 (300)		10 (600)		
Total			10 (600)		10 (600)		20 (1200)	

This interaction test will provide power to detect strong synergistic or antagonistic effects. It is a statistically efficient approach valuable for studying health care interventions, where rigorous evaluation is typically expensive [97].

#### Aim 1 Analyses

For aim 1, the outcome is a dichotomous measure of hemodialysis session stability. This outcome will be collected through digitally recorded blood pressures in all hemodialysis sessions of all patients at each facility who have not opted out of the study during the study period. For the continuous outcome measure of the proportion of unstable sessions, we will use linear mixed models to compare individuals in facilities in the 2 treatment arms to individuals in the nonintervention facilities. Random effects for facility clustering will be accounted for using the MIXED procedure in SAS (SAS Institute). As a sensitivity analysis, we will also examine hemodialysis session stability as a dichotomous (yes or no) variable and analysis will be carried out on the longitudinal data set using logistic regression with a random effect for both patients and facilities, using the GLIMMIX procedure in SAS. The random effects will model the correlations among the measurements at each level.

Three secondary analyses are planned for this aim. The first will test the treatment effects in a similar model but adding adjustment for any covariates found to be unbalanced between treatment groups in preliminary analyses. The next secondary analysis will test the interaction between the 2 interventions to detect a possible synergistic or antagonistic effect. The third will be a secondary analysis of patient proportion with IDH before versus after the intervention phase to test whether the intervention effects are maintained. The basic analysis is a paired comparison of percentage of IDH before versus after the intervention phase begins, with adjustments for length of intervention phase (24 wks) and follow-up phase (12 wks).

In additional sensitivity analyses, we will examine the effect of patient compliance and noncompliance rates (ie, the percentage of patients who accept peer mentoring and checklist adherence for provider intervention) on the results. The sensitivity analyses aimed to assess the robustness of the conclusions based on the analyses of the primary outcome, specifically to determine whether the findings hold under different methods of defining the primary outcome. Thus, we will conduct sensitivity analyses for the secondary measures of session instability described in *Data Collection* section. As part of this, because we will know the dates and causes of hospitalizations and death by cardiovascular- or fluid-related causes, we will identify those events that took place on days in which dialysis was administered. Accordingly, we will conduct a sensitivity analysis of the primary outcome of dialysis session stability both with, and without, those events on dialysis days included. For the primary outcome analyses, we will also conduct a sensitivity analysis without the sessions of patients who begin that session with a sitting predialysis SBP of 100 to 110 mm Hg and whether the sitting SBP falls below 90 mm Hg (using the lowest SBP during session) if starting SBP ≥100 mm Hg.

#### Aim 2 Analyses

Aim 2 incorporates 6 secondary outcome measures, which we will analyze at the individual level and summarize at the facility level. The interdialytic weight gain, symptom and quality of life analyses, based on continuous outcome measures, will be carried out using mixed models (the MIXED procedure of SAS). Note that the quality of life analyses will be based on data from 2 time points, one before intervention and one during the intervention, rather than session-specific data as analyzed for the other outcomes. Analyses of missed or shortened hemodialysis sessions will use logistic regression for longitudinal data. Analyses of hospitalizations and mortality will use logistic regression for the individual-level data. As part of these models, we will test IDH-related variables (eg, recent IDH and frequency of IDH in the past month) as potential predictors. In addition, we will conduct as-treated analyses for the peer mentoring intervention whereby only patients who received peer mentoring are compared with patients in the other treatment arms, with adjustment for the same factors as in the intent-to-treat analyses.

#### Aim 3 Analyses

Field notes compiled from provider training sessions 2 and 3, as well as the operations committee meetings, will be used to identify and understand barriers to implementing the interventions. Using NVivo (QSR International) qualitative data analysis software, we will also analyze data thematically [98] to identify and explain the types of implementation barriers and facilitators. We will use these qualitative analyses as supplementary explanations as we seek to explain the results of the trial, especially if there are differences in effectiveness between facilities implementing the same interventions. If necessary, modifications will be made while the trial is ongoing.

#### Heterogeneity of Treatment Effects and Mediation Analyses

The primary analysis will examine average treatment effects, but we will also examine the heterogeneity of treatment effects (HTE) on IDH rate for a variety of covariates, particularly those that are characterized by differential outcomes among patients on hemodialysis. Covariates of interest include demographics such as age, race [99], level of education [99], literacy, presence of comorbidities, and heart rate variability. Supporting these HTE analyses, greater benefits from peer mentoring for African Americans were found in a trial conducted by our advisory partner, the Michigan branch of the NKF [34]. Depending on the sample size obtained for each of the covariates of interest, either a subgroup analysis or an analysis including interaction terms will be used to examine HTE. Only two-way interactions between the treatment indicator and each of the covariates will be analyzed.

The second part of these analyses aims to identify mechanisms of action for the interventions, such as changes in patient or practitioner behavior and session characteristics; these changes in behavior will identify important pathways for improvement in reducing IDH. However, these analyses are not intended to be used for causal inferences. Each potential mediator (Textbox 2) will be tested separately, without adjusting for other mediators, although associations between mediators can be assessed separately for improved interpretation.

**Potential mediators of dialysis session stability.**

Patient-level variables

- Patient session instability prevention behavior
- Fluid management practice patterns
- Number and categories of medications patient is taking
- Fluid adherence (interdialytic weight gain)
- Dialysis adherence
- Patient symptoms (burden and recovery time)
- Hospitalizations for cardiovascular or fluid-related causes
- Quality of life

Session level variables

- Treatment time delivered
- Average session ultrafiltration rate
- Use of cool dialysate
- Use of sodium modeling
- Use of ultrafiltration modeling
- Ultrafiltration variability for the whole session, or until intradialytic hypotension incident
- Sitting-to-standing change in systolic blood pressure (SBP)
- Starting SBP
- Slope of changes in SBP over the whole session, or until intradialytic hypotension incident
- Values and times of lowest and highest SBP readings during session
- Patient request regarding fluid removal
- Fluid adherence (interdialytic weight gain)
- Previous achievement of postdialysis target weight
- Previous session instability

## Results

This study began enrolling participants in early 2020 but was paused during the COVID-19 pandemic. The study began in May 2022, and the first facilities began enrolling patients in January 2023. Intervention start dates will be staggered such that 4 facilities (1 from each intervention arm) start at once, while the next 4 facilities will begin preparation to join the study. The first 8 facilities will complete the intervention and follow-up data collection period in November 2023. The remaining 12 facilities will begin on a staggered timeline with follow-up data collection ending in November 2024.

## Discussion

### Principal Findings

The enhancement of the hemodynamic stability of hemodialysis is a critical concern for clinicians owing to study findings about the problem of hemodialysis-induced cardiovascular harm [20,100] and owing to vociferous calls for reform in fluid management practices in hemodialysis care [101-104]. A consensus statement from Medical Directors of United States hemodialysis facilities reflects this concern [22].

However, with a lack of clear guidance regarding hemodynamic stability enhancement, approaches to hemodynamic stability in hemodialysis care are variable. The current practice includes educating patients regarding appropriate fluid intake, individualized treatment targets and fluid removal goals, regular monitoring and assessment during dialysis treatments, regular medication reviews, intervening as IDH emerges, and management of emergent symptoms. However, there are also wide facility-level variations in IDH rates, demonstrating the ability of practice patterns to influence cardiovascular session stability. For instance, IDH prevalence varied between 11.1% and 25.8% in a study of 13 US facilities [11]. In that study, it was demonstrated that facility was also a significant predictor of IDH with odds ratios between 0.608 and 1.468 after adjustment for patient characteristics.

Although the high prevalence of dialysis instability demands change, hemodialysis providers lack critical guidance for preventing IDH in usual practice. Our evidence-based approach contends that to prevent unstable hemodialysis sessions, optimal fluid management must be at the forefront of typical care practices. In line with a growing body of evidence, we support gentle adjustment of posthemodialysis target weights and prolongation of treatment time prescriptions, regular assessment of patient fluid volume status, lower UFR (preferably <10-12 mL/kg/hr), and early intervention if a patient begins to show signs of instability. We do so while continuing to emphasize lower interdialytic weight gain and a low-sodium diet to reduce thirst, and thus fluid intake [83,105-109]. We also promote use of interventions such as use of cool dialysate, blood pressure medication review, and evaluation of patients for home dialysis, as appropriate (Figure 1). To facilitate this fluid management–focused practice, our intervention translates successful patient safety interventions and patient behavior interventions from inpatient and chronic disease care settings, respectively, to the outpatient hemodialysis context.

### Novelty of This Study

Although checklists for patient safety have been growing in popularity in health care organizations in the United States and internationally, they have primarily been used in inpatient settings, particularly surgery [110-112] and critical care [113,114]. However, a recent trial on a checklist for hemodialysis care infection control [115,116] and another recent pilot in Canada [117] illustrated that hemodialysis care is an outpatient context that is well suited for checklist implementation. When combined with staff training, our novel application of checklists to hemodialysis care makes it an innovative and promising approach to care improvement.

Patient involvement in safety is encouraged by national and international stakeholder organizations [118-121]. Patient involvement is particularly promising in the context of efforts to improve hemodialysis session stability because patients already make daily decisions such as how much sodium and fluid to consume and whether to shorten or skip hemodialysis sessions—often without realizing the corresponding cardiovascular and hemodynamic stability implications. However, few studies to date have examined the potential for patient involvement in the prevention of hemodialysis session instability as a complication of hemodialysis care [119,122].

Outside hemodialysis care, practices designed to encourage patient involvement in the prevention of health care complications have seen mixed success in producing behavior change [123]. This is likely because of lack of education and support in prior initiatives [124-126]. Furthermore, prior efforts have been rarely grounded in validated theories of behavior change [123], relying instead on simple information provision, such as personalized care records [127] and verbal or written instruction [128-130]. Rarely have interventions explicitly used health behavior change theory, a component of our evidence-based intervention that increases its likelihood of success. Furthermore, although multimodal engagement strategies have been advocated [131], no intervention trial has examined the potential for patients to assist each other in preventing health care complications*.*

Supporting a peer-based approach to promoting hemodialysis session stability, there has been growing interest in peer-based health care approaches as trials have shown benefits in health outcomes [125-128] and behavior [129-131]—including complex health behaviors relevant to IDH, such as diet change [128,132]. Therefore, through an innovative application of peer mentoring, we aim to support the cardiovascular and hemodynamic stability of hemodialysis by helping patients become more actively engaged in treatment and self-care decisions that affect their cardiovascular well-being. We will address patients’ frequent lack of information to help them meaningfully participate in hemodynamic stability–related decisions such as fluid removal targets, session length, and frequency. Our preliminary studies showed that some patients already try to influence their treatment targets and speed to reduce distressing symptoms and that patients with ESKD share strategies for making hemodialysis more tolerable [132]. Trained peer mentors are in an excellent position to provide this information systematically and in a manner that is accessible, actionable, and influential. Thus, our innovative approach meets important patient needs while enhancing the likelihood of success in mobilizing patients to engage in behaviors that will increase hemodialysis session stability.

### Study Limitations

The study is being conducted in the United States in collaboration with one large hemodialysis facility chain. Although this may limit generalizability, we note that hemodialysis care in the United States is predominately provided by 2 large facility chains, one of which is a study partner. This study partner has >2000 facilities, which suggests the potential for substantial reach if the intervention proves successful. Study facilities were stratified by region and county poverty level, ensuring representation of facilities from 4 US regions and varied patient populations in the study. Furthermore, this study, as with other pragmatic trials, is dependent on care providers’ data entry as part of routine care. In an effort to support completeness of study data, some training of staff in data collection will be undertaken as part of the facility staff onboarding process. This study will leverage a robust data infrastructure used for ongoing clinical trials.

### Conclusions

The effects of provider and patient education on patient-centered clinical outcomes will be analyzed, and the findings will be used to inform further improvements in patient care. Improving the hemodynamic stability of hemodialysis sessions is a critical concern for clinicians and patients, and educating providers and patients is predicted to lead to improvements in patient health outcomes and quality of life.

## Appendix

Multimedia Appendix 1 Patient intervention interactive modules—general website design.

Multimedia Appendix 2 Patient intervention interactive modules—educational module design.

Multimedia Appendix 3 Peer mentoring satisfaction surveys.

Multimedia Appendix 4 Practice patterns surveys.

Multimedia Appendix 5 Operations committee discussion questions.

Multimedia Appendix 6 Peer review report from PCORI Funding Announcement: Large Pragmatic Studies to Evaluate Patient-Centered Outcomes - Improving Healthcare Systems (Patient-Centered Outcomes Research Institute, USA).

## Abbreviations

ESKD: end-stage kidney disease
HIPAA: Health Insurance Portability and Accountability Act
HTE: heterogeneity of treatment effects
IDH: intradialytic hypotension
IRB: institutional review board
NKF: National Kidney Foundation
SBP: systolic blood pressure
UFR: ultrafiltration rate

## Data Availability

The data sets generated during or analyzed during this study are not publicly available because of concerns about patient identifiability and confidentiality. The patient information sheet provided to patients at the outset of the study clarifies that their information will not be shared in a raw form with anyone except the University of Michigan research team.
