# Supplementary figures and images for: Enhancing the Cardiovascular Safety of Hemodialysis Care Using Multimodal Provider Education and Patient Activation Interventions: Protocol for a Cluster Randomized Controlled Trial
Source: JMIR Res Protoc. 2023 Apr 20;12:e46187. doi: 10.2196/46187 (PMC10160944; doi:10.2196/46187)

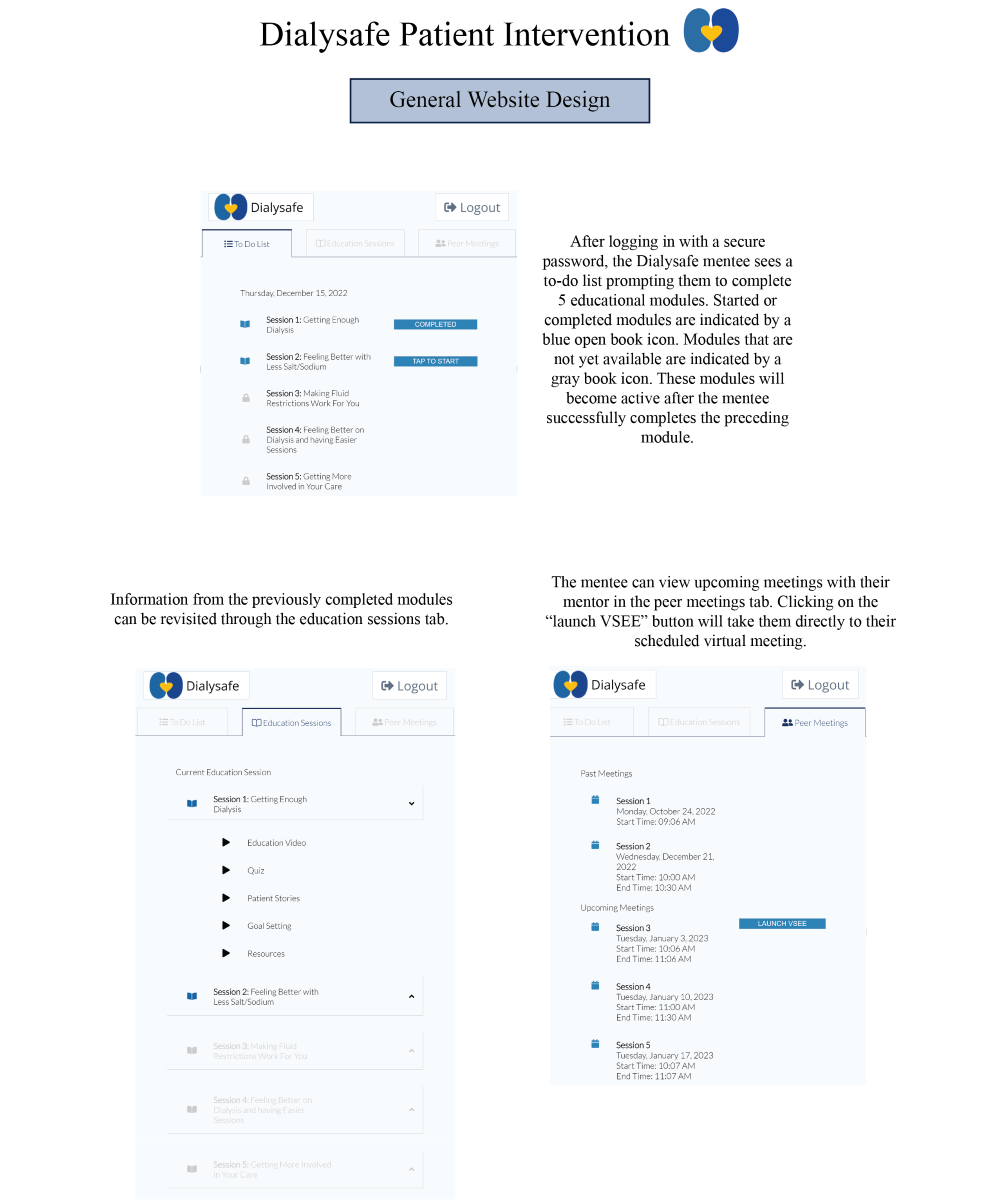

Supplement: Multimedia Appendix 1 [file resprot_v12i1e46187_app1.png]

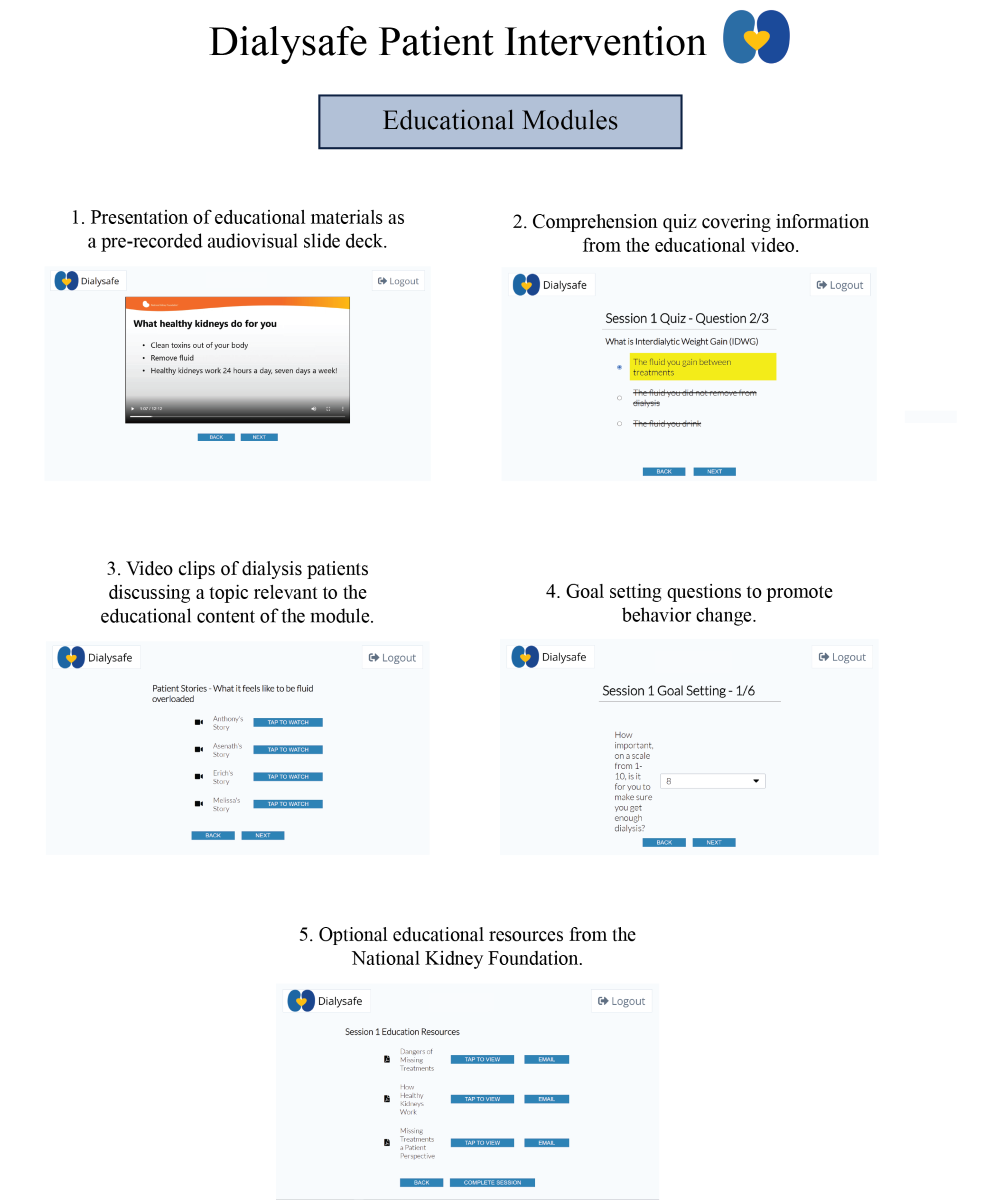

Supplement: Multimedia Appendix 2 [file resprot_v12i1e46187_app2.png]
