# Supplementary material for: Enhancing the Cardiovascular Safety of Hemodialysis Care Using Multimodal Provider Education and Patient Activation Interventions: Protocol for a Cluster Randomized Controlled Trial
Source: JMIR Res Protoc. 2023 Apr 20;12:e46187. doi: 10.2196/46187 (PMC10160944; doi:10.2196/46187)
Supplement: Multimedia Appendix 3 [file resprot_v12i1e46187_app3.docx]

***Mentee Satisfaction Survey***

1. Overall how would you rate your experience with the Dialysafe peer mentoring program?

- Excellent
- Good
- Fair
- Poor

1. Please tell us how much you agree or disagree with the following statements: (1-5 Strongly Agree to Strongly Disagree)

- My mentor was helpful
- My mentor was supportive
- My mentor was knowledgeable
- The tablet system was easy to use to talk to my mentor.

1. How easy or hard was it for you to get the tablet to use in your Peer Mentoring Sessions?

(1-5; very hard to very easy)

1. How easy or hard was it for you to return the tablet to Study Staff at the University of Michigan?

(1-5; very hard to very easy)

1. Please tell us how much you agree or disagree with the following statements: (1-5 Strongly Agree to Strongly Disagree)

- I think that I would like to use the tablet frequently.
- I found the tablet unnecessarily complex.
- I thought the tablet was easy to use.
- I needed the support of another person to be able to use the tablet.
- I found that the various functions in the tablet were well integrated.
- I thought there was too much inconsistency in the tablet.
- I would imagine that most people would learn to use the tablet very quickly.
- I found the tablet very cumbersome to use.
- I felt very confident using the tablet.
- I needed to learn a lot of things before I could get going with the tablet

1. Did you share the paper titled “Patient Peer Mentoring Program: Information for Family Members of Participants” (pictured right) with anyone? (Yes/No)
   1. If yes, how many people did you share it with? __
   2. If yes, who did you share it with? (Check all that apply)
      - Spouse/partner
      - Adult child/children
      - Parent(s)
      - Sibling(s)
      - Niece(s) or nephew(s)
      - Cousin(s)
      - Friend(s)
      - Other ______
2. Please tell us how much you agree or disagree with the following statements: (1-5 Likert: Strongly Disagree to Strongly Agree)

During the time I have been receiving peer mentoring:

- My family has really tried to help me.
- I got the emotional help and support I needed from my family.
- I could talk about my problems with my family.
- My family was willing to help me make decisions.
- My family was willing to help me reach my goals.

1. Did you tell any other patients in your clinic about things you learned from the educational or peer mentoring sessions? Yes/No

- If yes, how many people did you tell? __

1. Did any other patients in your clinic tell you about things they learned from the educational or peer mentoring sessions? Yes/No

- If yes, how many people told you things? __

1. I would recommend the Dialysafe peer mentoring program to someone else in a similar situation. (1-5 Strongly Agree to Strongly Disagree)
2. Did you make changes, or do anything differently, in these areas as a result of the Dialysafe peer mentoring program? (check all that apply)
   - Limiting my sodium intake
   - Tracking my sodium intake
   - Limiting my fluid Intake
   - Tracking my fluid intake
   - Reporting symptoms during my dialysis session to dialysis staff
   - Tracking my weight at home
   - Tracking my blood pressure at home
   - Talking to my dialysis team about my estimated dry weight
   - Talking to my dialysis team about my fluid removal goal or ultrafiltration rate
   - Not shortening or skipping dialysis sessions
   - Shortening or skipping fewer sessions
   - Attending extra, or longer, dialysis sessions as needed
   - Attending my care plan meeting at my facility
   - Keeping my care team informed about changes in my health such as emergency room trips, upcoming procedures, or medication changes
3. Did you feel the Dialysafe program helped you set goals? Yes/No
4. Did the Dialysafe program help you reach the goals you set? Yes/No
5. Was it helpful to discuss goals with your mentor after each educational session? Yes/No
6. What did you like best about the Dialysafe program? (Open text)
7. How could we improve the Dialysafe program? (Open text)

***Mentor Satisfaction Survey***

1. Overall how would you rate your experience with the Dialysafe program?

- Excellent
- Good
- Fair
- Poor

Please tell us how much you agree or disagree with the following statements about your experience with your mentee:

1. The program allowed me to be helpful to my mentee(s). (1-5 Strongly Agree to Strongly Disagree)
2. I had a good relationship with my mentees (1-5 Strongly Agree to Strongly Disagree)
3. My mentee(s) and I could trust each other. (1-5 Strongly Agree to Strongly Disagree)
4. My mentee(s) made positive changes to their health because of our time together. (1-5 Strongly Agree to Strongly Disagree)

Please tell us how much you agree or disagree with each of these statements about your personal experience with the program:

1. The National Kidney Foundation staff is supportive to peer mentors. (1-5 Strongly Agree to Strongly Disagree)
2. The National Kidney Foundation staff is available to help when needed. (1-5 Strongly Agree to Strongly Disagree)
3. I would recommend becoming a peer mentor for the Dialysafe program to a friend. (1-5 Strongly Agree to Strongly Disagree)
4. I would be willing to be a mentor in a program like Dialysafe again in the future. (1-5 Strongly Agree to Strongly Disagree)
   1. Please explain why you would/would not want to be part of a similar program.

Please answer the following questions about peer mentor training.

1. After peer mentor training, I felt prepared to discuss topics regarding intradialytic hypotension with a mentee.
   1. Strongly Agree to Strongly Disagree)
2. After peer mentor training, I felt prepared to use the web-based management system.

(1-5 Strongly Agree to Strongly Disagree)

1. What, if anything, did you find most helpful about the peer mentor training? (Open text)
2. What, if anything, would you change about the peer mentor training? (Open text)

Please answer the following questions about the web page you used to participate in Dialysafe. How much do you agree or disagree with the following statements?

1. The web-based management system was easy to use to connect with my mentee(s).

(1-5 Strongly Agree to Strongly Disagree)

1. The web-based management system was easy to use to look at session materials.

(1-5 Strongly Agree to Strongly Disagree)

1. The web-based management system was easy to use to complete the topic checklist after meeting with my mentee(s).

(1-5 Strongly Agree to Strongly Disagree)

1. The web-based management system was easy to use for filling in my mentee’s action plan.

(1-5 Strongly Agree to Strongly Disagree)

Now we’re going to ask you some questions about your health.

How much do you agree or disagree with the following statements?

1. Being a mentor in Dialysafe taught me more about my own health. (1-5 Strongly Agree to Strongly Disagree)
2. I noticed that I changed my own health behaviors based on what I talked about with my mentee. (1-5 Strongly Agree to Strongly Disagree)
3. Did you make changes, or do anything differently, in these areas because of the Dialysafe program?

|  | Yes | No | I don’t know | Not Applicable |
| --- | --- | --- | --- | --- |
| Diet |  |  |  |  |
| Exercise |  |  |  |  |
| Fluid Intake |  |  |  |  |
| Dialysis Session Attendance |  |  |  |  |
| Care Plan Meeting Attendance |  |  |  |  |

Please share your final thoughts in the questions below:

1. What did you like best about the Dialysafe program? (Open text)
2. How could we improve the Dialysafe program? (Open text)
