# Supplementary material for: Enhancing the Cardiovascular Safety of Hemodialysis Care Using Multimodal Provider Education and Patient Activation Interventions: Protocol for a Cluster Randomized Controlled Trial
Source: JMIR Res Protoc. 2023 Apr 20;12:e46187. doi: 10.2196/46187 (PMC10160944; doi:10.2196/46187)
Supplement: Multimedia Appendix 4 [file resprot_v12i1e46187_app4.docx]

***Practice Patterns Survey: Nurse Managers***

Purpose of this Survey and Broad Guidelines for its Completion

This survey is intended to assess the "typical" practice or policies at your dialysis facility with respect to fluid management and prevention and treatment of intradialytic hypotension. It is not a knowledge test.

All questions should be answered regarding patients receiving in-center-based hemodialysis only.

This survey should take approximately 10 minutes to complete.

Question Block 1:

First, we'd like to know some information about you and your hemodialysis facility.

1. What is your job title?
2. What is the name of your hemodialysis facility?
3. In what state is your hemodialysis facility located?
4. In what city/town is your hemodialysis facility located?
5. Does your facility have one or more isolation shift(s) for patients with confirmed or suspected COVID-19?
   - Yes
   - No
6. How many COVID-19 isolation shifts does your facility have?
7. How often are patients seen by nephrologists during rounds in your facility?
   - Daily
   - 2-4 times a week
   - Once a week
   - Every 2 weeks
   - Every 3 weeks
   - Monthly
   - Other (please describe) ______________
   - I do not know
8. How often are patients seen by advanced practitioners (e.g., Advance Practice Nurse, Specialist Nurse Practitioner, Physician Assistant, etc.) during rounds in your facility?
   - Daily
   - 2-4 times a week
   - Once a week
   - Every 2 weeks
   - Every 3 weeks
   - Monthly
   - Other (please describe) ______________
   - There are no advanced practitioners who round at this facility
   - I do not know

Question Block 2

Now we’re going to ask you about practices for the determination of patients’ estimated dry weight (EDW) and for the assessment of patients’ volume/fluid status in your facility.

1. For a typical patient in your dialysis facility, how often is EDW assessed?
   - Each treatment
   - Weekly
   - Monthly
   - Quarterly
   - Twice a year
   - Yearly
   - No specific frequency. Done as clinically indicated.
   - Other (please describe) ____________
   - I do not know
2. At your facility, who identifies when the EDW of a patient should be reviewed? (Select all that apply)
   - Nephrologist
   - Advanced practice provider (ANP, NP or PA)
   - Dialysis Nurse
   - Patient care technician
   - Dieticians
   - Social workers
   - Designated fluid manager at the facility. Please specify the type of healthcare practitioner that occupies the fluid manager role: _______________
   - Other (please specify) ______________
   - I do not know
3. Some facilities designate a “fluid manager” as locally responsible for volume/fluid management issues. How often is a “fluid manager” involved in volume/fluid management for patients in your facility?
   - There is no fluid manager role at my facility
   - Each treatment
   - Weekly
   - Monthly
   - Quarterly
   - Twice a year
   - Yearly
   - No specific frequency. Done as clinically indicated.
   - Other (please specify) ____________
   - I do not know
4. How often is an approved algorithm or protocol used to assess patient volume/fluid status in patients at your facility?
   - There is no such approved algorithm or protocol at my facility
   - Each treatment
   - Weekly
   - Monthly
   - Quarterly
   - Twice a year
   - Yearly
   - No specific frequency. Done as clinically indicated.
   - Other (please specify) ____________
   - I do not know
5. How often is the facility dietician actively involved in volume/fluid management for patients at your facility?
   - Never
   - Each treatment
   - Weekly
   - Monthly
   - Quarterly
   - Twice a year
   - Yearly
   - No specific frequency. Done as clinically indicated.
   - Other (please specify) _______________
   - I do not know

Question Block 3

The following questions are designed to gauge the extent of patients’ awareness of, and involvement in, volume/fluid management at your facility.

1. Are patients at your facility encouraged to monitor their weight at home on a regular basis?
   - Yes
   - No
   - I do not know
2. Are patients at your facility encouraged to tell a staff member whether they believe their estimated dry weight (EDW) is appropriate for them?
   - Yes
   - No
   - I do not know
3. Are patients at your facility encouraged to report symptoms suggestive of fluid overload at each hemodialysis session?
   - Yes
   - No
   - I do not know
4. Are patients at your facility encouraged to report symptoms suggestive of intradialytic hypotension at each hemodialysis session?
   - Yes
   - No
   - I do not know
5. Are patients at your facility told about the importance of checking both sitting and standing blood pressure?
   - Yes
   - No
   - I do not know

Question Block 4

Now we’re going to ask you about the speed of fluid removal during patient sessions, and how you prevent and manage intradialytic hypotension (IDH) in your facility.

1. Who determines target ultrafiltration rates (UFRs) for patients at your dialysis facility? (choose all that apply)
   - Nephrologist
   - Physician extenders (NP or PA)
   - Dialysis Nurse
   - Patient care technician
   - Designated fluid management “champion” at the facility
   - Other (please specify) _____________
2. Which of the following are administered per approved algorithm or protocol (with appropriate physician order) for the management of intradialytic hypotension? (mark all that apply)
   - IV normal saline
   - Trendelenburg position
   - Slowing/stopping ultrafiltration
   - Prolongation of treatment time (without isolated UF)
   - Provision of extra hemodialysis session
   - IV hypertonic saline
   - Dextrose infusion
   - Decreased blood flow (pump speed)
   - Cool dialysate
   - I do not know
   - My facility does not have protocol for the management of IDH
3. How is your dietician involved in preventing IDH? (check all that apply)
   - By providing routine counseling on sodium and fluid management for all dialysis patients
   - By advising patients on the relationship between IDH and fluid management
   - By reviewing blood pressure changes during previous dialysis sessions
   - By providing intensive counseling on sodium restrictions for patients with recurrent IDH
   - By providing intensive counseling on fluid restrictions for patients with recurrent IDH
   - By advising patients on the dangers of IDH
   - They are not involved in prevention or management of IDH
   - Other (please specify) ________________
   - I do not know
4. Are you aware of the availability of the Fluid Management Dashboard within your facility?
   - Yes
   - No
5. Is the Fluid Management Dashboard used during your monthly quality meetings?
   - Always
   - Often
   - Sometimes
   - Rarely
   - Never
6. Do the attending physicians, NPs and Pas use the Fluid Management Dashboard to identify patients with fluid management problems?
   - Always
   - Often
   - Sometimes
   - Rarely
   - Never
7. Other than training provided by the Dialysafe program, have staff at your clinic been provided with fluid management-focused training in the past year?
   - Yes
   - No
   - I do not know
8. How many times did staff at your clinic receive fluid management-focused training in the past year?
   - 1
   - 2
   - 3
   - 4
   - 5
   - More than 5
9. When did staff at your clinic receive this training?

|  | Please include date or leave blank if not applicable | |
| --- | --- | --- |
|  | Month | Year |
| Training 1 |  |  |
| Training 2 |  |  |
| Training 3 |  |  |
| Training 4 |  |  |
| Training 5 |  |  |

We thank you for your time spent taking this survey.

Your response has been recorded.

***Practice Patterns Survey: Medical Directors***

Purpose of this Survey and Broad Guidelines for its Completion

- This survey is to be completed in conjunction with your facility’s involvement in the Dialysafe study.
- This survey is not designed to be a knowledge test, but is intended to assess the “typical” practice or policies in your dialysis facility with respect to fluid-weight management and prevention and treatment of intradialytic hypotension.
- All questions should be answered with regards to patients receiving in-center-based hemodialysis only.
- This survey will take approximately 10 minutes to complete.

Question Block 1

First, we’d like to know some information about you and your hemodialysis facility.

1. What is your job title?
2. What is the name of your hemodialysis facility?
3. In what state is your hemodialysis facility located?
4. In what city/town is your hemodialysis facility located?

Question Block 2

Now we’re going to ask you about practices for the determination of patients’ estimated dry weight (EDW) in your facility.

1. For a typical patient in your dialysis facility, how often is EDW usually assessed?
   - Each treatment
   - Weekly
   - Monthly
   - Quarterly
   - Twice a year
   - Yearly
   - No specific frequency. Done as clinically indicated.
   - Other (please describe) _______________
   - I do not know
2. How often is a formal protocol used to assess EDW in patients in your facility?
   - Each treatment
   - Weekly
   - Monthly
   - Quarterly
   - Twice a year
   - Yearly
   - No specific frequency. Done as clinically indicated.
   - Other (please describe) _______________
   - I do not know
3. How often are the following patient-related factors considered when assessing a patient’s EDW in your facility?

Select one:

| Never | Each Month | Weekly | Monthly | Quarterly | Twice a year | Yearly | No specific frequency. Done as clinically indicated | I do not know |
| --- | --- | --- | --- | --- | --- | --- | --- | --- |
| օ | օ | օ | օ | օ | օ | օ | օ | օ |

- Patient’s home blood pressure (BP)
- Patient’s home weight monitoring
- History of intradialytic hypotension (IDH)
- Patient’s own assessment of their optimal EDW
- Patient-reported muscle cramps during dialysis
- Patient-reported post-dialysis recovery time or fatigue
- Patient skipping and/or shortening dialysis treatments
- Patient’s interdialytic weight gain
- Patient’s residual renal function
- Review and reconciliation of blood pressure related medications with patient

1. How often are the following techniques used to determine a patient’s EDW in your facility?

Select one:

| Never | Each Month | Weekly | Monthly | Quarterly | Twice a year | Yearly | I do not know |
| --- | --- | --- | --- | --- | --- | --- | --- |
| օ | օ | օ | օ | օ | օ | օ | օ |

- Physical examination
- Orthostatic blood pressure measurement
- Chest X-ray
- Multi-frequency bioimpedance device
- Single-frequency bioimpedance device
- Online relative blood volume monitoring to determine target weight (i.e., for diagnostic purposes), using Crit-Line® or other device
- Online relative blood monitoring using a biofeedback device for machine-driven regulation of fluid removal (i.e., for therapeutic purposes), using Hemocontrol™ or other device

1. “Dry weight probing” is a method of identifying a patient’s optimal EDW by reducing their target weight in progressive, small increments (e.g., 0.2 - 0.3 kg). How often is dry weight probing applied in your facility?
   - Never
   - Occasionally
   - Some of the time
   - In new patients only
   - Only in patients in which it is difficult to achieve desired target weight
   - Post hospitalization
   - In majority of patients
   - In all patients
   - I do not know

Question Block 3

Now we’re going to ask you about specific volume/fluid management policies and roles that you may have in your facility.

1. How often is a formal protocol used to assess patient volume/fluid status in patients in your facility?
   - There is no such formal protocol at my facility
   - Each treatment
   - Weekly
   - Monthly
   - Quarterly
   - Twice a year
   - Yearly
   - No specific frequency. Done as clinically indicated.
   - Other
   - I do not know
2. Some facilities designate a “fluid manager” as locally responsible for volume/fluid management issues. How often is a “fluid manager” involved in volume/fluid management for patients in your facility?
   - There is no fluid manager role at my facility
   - Each treatment
   - Weekly
   - Monthly
   - Quarterly
   - Twice a year
   - Yearly
   - No specific frequency. Done as clinically indicated.
   - Other
   - I do not know
3. How often is the facility dietician actively involved in volume/fluid management for patients in your facility?
   - Never
   - Each treatment
   - Weekly
   - Monthly
   - Quarterly
   - Twice a year
   - Yearly
   - No specific frequency. Done as clinically indicated.
   - Other
   - I do not know
4. Does your facility have a home blood pressure monitoring policy for patients?
   - Yes
   - No
   - I do not know

Question Block 4

Now we’re going to ask you about the treatment times of patients in your facility.

1. What is the usual session length for **new patients** starting thrice-weekly hemodialysis session in your facility?
   - 2 hours or less for most
   - 3 hours or less for most
   - 3 hours for most
   - 3.5 hours for most
   - 4.0 hours for most
   - More than 4.0 hours for most
   - The shortest time necessary to achieve target Kt/V for most
   - I do not know
2. What is the usual treatment time for **ongoing patients** on thrice-weekly hemodialysis in your facility?
   - 3 hours or less for most
   - 3 hours for most
   - 3.5 hours for most
   - 4.0 hours for most
   - More than 4.0 hours for most
   - The shortest time necessary to achieve target Kt/V for most
   - I do not know

Question Block 5

Now we’re going to ask you about how you prevent and manage intradialytic hypotension (IDH) in your facility.

1. Are the following practices used for the management of excessive interdialytic weight gain in patients in your facility?

Select one:

| No | Yes, routinely | Yes, infrequently | I do not know |
| --- | --- | --- | --- |
| օ | օ | օ | օ |

- Prolongation of treatment time (without isolated ultrafiltration)
- Sequential dialysis and ultrafiltration
- Extra hemodialysis session
- Lowering dialysis sodium
- Lowering dietary sodium as a primary strategy
- Lowering fluid intake as a primary strategy
- Improving glycemic control in diabetics
- Intensive dietary counseling especially about eating out and tips for home cooking
- Changing medication to avoid side effects resulting in high fluid intake (e.g., those causing dry mouth)
- Intensive social work input for missed/shortened sessions

1. For patients you are prone to IDH, how important are the following management strategies in your facility?

Select one:

| Not at all important | Slightly important | Neither important nor unimportant | Moderately important | Very important | I do not know |
| --- | --- | --- | --- | --- | --- |
| օ | օ | օ | օ | օ | օ |

- EDW review
- Progressive, stepwise challenging of estimated dry weight in small increments
- Standing protocols for patient (i.e., maximum fluid removal, blood pressure or heart rate cutoffs)
- Lower ultrafiltration rate
- Promotion of dietary sodium restriction
- Extra dialysis session
- Lengthening treatment time to 4 hours or longer (if they were less than 4 hours prior)
- Change patient to 4 times per week dialysis regimen
- Lower dialysate temperature
- Avoidance of anti-hypertensive meds prior to dialysis
- Review current antihypertensive medication prescriptions
- Sequential dialysis and ultrafiltration
- Pre-emptive placement of patient in modified Trendelenburg position
- More frequent blood pressure monitoring during session (e.g., every 15 minutes)
- Online volume indicator, without automatic feedback regulation
- Online volume indicator, with automatic feedback regulation (e.g., automated changes to UF rate or dialysate conductivity)
- Sodium modeling/profiling
- Refer patients to home dialysis modalities

1. How often is your facility dietician involved in preventing IDH?
   - Not at all
   - Occasionally
   - On an as needed basis
   - Only in difficult to manage patients
   - They follow blood pressure changes during dialysis in all patients but become involved in prevention only in those that have recurrent IDH
   - They advise all patients on the dangers of IDH and are integral to its prevention
   - Other (please describe) ___________________
   - I do not know

Question Block 6

Now we’re going to ask you some questions about the amount and speed of fluid removal during patient hemodialysis sessions in your facility.

1. What is your facility’s limit on the total amount of fluid removed during a single dialysis session?
   - My facility does not have a policy that limits the amount of fluid removed during a single dialysis session
   - 4,000 mL
   - 4,500 mL
   - 5,000 mL
   - 5,500 mL
   - 6,000 mL
   - > 6,000 mL
   - Individual patients limit
   - Other (please specify) ________________
   - I do not know
2. What is your facility’s limit on the amount of fluid removed per hour (not including isolated ultrafiltration)?
   - My facility does not have a limit on the amount of fluid removed per hour
   - 800 mL/hour
   - 900 mL/hour
   - 1,000 mL/hour
   - 1,100 mL/hour
   - 1,200 mL/hour
   - > 1,200 mL/hour
   - I do not know
3. What is your facility’s target maximum UFR?
   - There is no target maximum UFR in my facility
   - 8 ml/kg/hour
   - 10 ml/kg/hour
   - 13 ml/kg/hour
   - 15 ml/kg/hour
   - 20 ml/kg/hour
   - I do not know

You will receive a $250 honorarium for completing this survey. In order to facilitate payment, please type full name below. Your name will be deleted from our dataset once your honorarium has been issued.

1. What is your full name?

We thank you for your time spent taking this survey.

Your response has been recorded.
