# Supplementary material for: Enhancing the Cardiovascular Safety of Hemodialysis Care Using Multimodal Provider Education and Patient Activation Interventions: Protocol for a Cluster Randomized Controlled Trial
Source: JMIR Res Protoc. 2023 Apr 20;12:e46187. doi: 10.2196/46187 (PMC10160944; doi:10.2196/46187)
Supplement: Multimedia Appendix 5 [file resprot_v12i1e46187_app5.docx]

**Agenda for Operations Committee Meetings**

1. **Introductions/attendance**
2. **State of study updates**

- At pre-intervention operations committee meeting:
  - Enrollment rates
- At the two operations committee meetings during the intervention period:
  - Intervention status
    - *As applicable:*
      - Peer mentoring recruitment, retention and educational session completion
      - IDH prevention completion rates
      - Provider training completion rates

1. **Questions for patient representatives**
   - What is your overall impression of the study so far?
   - Have you noticed any positive or negative changes in dialysis treatments (in yourself or other patients) since the study began?
   - Do you have any concerns about the study?
   - Problem solving/troubleshooting, if needed
2. **Questions posed to staff after patient representatives have left the meeting**

- At pre-intervention operations committee meeting:
  - Provider intervention facilities
    - We are planning to train you to use a checklist to identify patients at risk of intradialytic hypotension. The checklist will rely on information from the fluid management tab. Is there anything that we should consider about how you use the fluid management tab now that we should take into account in planning your training?
- At the two operations committee meetings during the intervention period:
  - Provider intervention facilities
    - Your facility has been given a checklist to use to identify patients at increased risk of IDH. Please tell us about how you are using the checklist.
      - *Follow-up questions as needed:*
        - Who is completing the checklist?
        - When are they completing the checklist?
        - What do they do before completing the checklist?
        - What do they do after completing the checklist?
      - What is working well about using the checklist?
      - Are there things that are not working well with using the checklist? If so, what are they?
  - Patient intervention facilities
    - Some patients at your facility have been given tablet computers for use at home to obtain peer mentoring and education about IDH prevention.
      - *Follow-up questions as needed:*
        - Have patients brought their tablet computers into the clinic? If so, please tell us what happened.
        - Have patients talked to any of you about anything regarding tablet use?
        - Have you noticed any changes in topics that patients are bringing up in their appointments with you?
        - Has anyone started to record things at home and bring in data and do you have reason to believe it’s because of the intervention?
        - Have you noticed patients talking to each other about the intervention?

1. **Problem solving, if needed**
2. **Questions/comments/concerns**
3. **Next steps and wrap up**
