# Supplementary material for: Enhancing the Cardiovascular Safety of Hemodialysis Care Using Multimodal Provider Education and Patient Activation Interventions: Protocol for a Cluster Randomized Controlled Trial
Source: JMIR Res Protoc. 2023 Apr 20;12:e46187. doi: 10.2196/46187 (PMC10160944; doi:10.2196/46187)
Supplement: Multimedia Appendix 6 [file resprot_v12i1e46187_app6.pdf]

PATIENT-CENTERED OUTCOMES RESEARCH INSTITUTE
SUMMARY STATEMENT
(Privileged Communication)

**Principal Investigator:**	Tiffany Veinot
**Organization:**	University of Michigan
**Project Title:**	Enhancing the cardiovascular safety of hemodialysis care: a cluster-randomized, comparative effectiveness trial of multimodal provider education and patient activation interventions
**PCORI Funding Announcement:**	Improving Healthcare Systems
**Review Cycle:**	Spring 2015 Cycle
**Request ID:**	SC15-1503-27848

AVERAGE OVERALL SCORE: 32

QUARTILE: 1

In-Person Review Discussion Notes:

Strengths:

-The focus illness, chronic kidney disease, is prevalent with high morbidity and mortality.

-The results have strong potential to meaningfully improve the delivery of care for affected patients.

-The study results are actionable and have the potential to be widely disseminated to dialysis clinics.

-There is a good conceptual framework and the measures selected are appropriate.

-The investigative team is strong with impressive interdisciplinary representation.

-There is team training for doctors, and patient activation measures and peer mentors for patients.

-Reviewers appreciated the emphasis of the patient role in the patient safety aspects of the dialysis session.

-Patient engagement is strong. The investigative team is partnering with the National Kidney Foundation, with trained peers to deliver the intervention

-The investigative team has secured the commitment of Fresenius dialysis centers, a major dialysis provider.

Weaknesses:

-Reviewers asked for more detail about the patient's role post-intervention.

-The estimated 70% participation rate may be overly optimistic given characteristics of this population, as this is a complex and very sick population, with a 15 % mortality rate. The difficulties of recruiting and retaining such patients throughout the study is not well addressed.

-The 2x2 study design does not appear to be reflected in the proposed analyses. The plan is to test each intervention arm versus no intervention, and may not be adequately powered to compare the two intervention arms to each other.

-The study appears to be powered only for the primary outcome variable, hypotension, which may lead to insufficient power for other important study variables.

-The estimated effect size, a 20% decrease in the outcome measure, may be overly optimistic.

-Reviewers expressed concern that differential attrition rates among study arms may create an imbalance in the study design. The application should discuss this issue and how to handle these missing data, particularly for mortality and patients whose ill health may require them to be removed from the analysis.

-While oversight committees at each study site is a strength, reviewers questioned whether there is a system in place for sites to communicate with each other.

The following reviewer critiques were completed prior to the in-person review and were not altered post-discussion.

Criterion 1: Impact of the condition on the health of individuals and populations

<u>Reviewer 1</u>:

Strengths:

-The application addresses a prevalent problem in the US. Chronic kidney disease (CKD) is a major and growing cause of death and disability in the US population.

-Patients with chronic kidney disease who are on hemodialysis are likely to also suffer from other chronic disease states, such as cardiovascular disease or diabetes.

-Among patients on hemodialysis, session instability, which is associated with cardiovascular harm, affects up to 20% of the patients. Session instability has a significant effect on patient quality of life.

-To a certain degree, session instability is potentially modifiable or preventable by adherence to diet and a treatment regimen.

Weaknesses:

-None noted.

Reviewer 2:

Reviewer 3:

Reviewer 4:

Strengths:

- End-stage renal disease (ESRD) is a tremendous burden on patients, families, and the health care system. Nearly half a million Americans are on dialysis.
- Hemodialysis (HD) to peritoneal dialysis (PD) is approx. 10:1. Transplantation occurs in <10%. There are >6000 HD facilities.
- ESRD leads to hypertension, coronary disease, heart failure, and sudden cardiac death (SCD); cardiovascular disease (CV) is the major cause of death.
- Hemodialysis session instability (e.g., "bad runs," hypotension) is common, and related to patient and provider factors.

Weaknesses:

- The link between HD instability and major CV events is controversial in its magnitude. The association between HD instability and outcomes is at least partly if not largely associative – sicker patients have more HD instability. The authors state at one point that 20% is due to patient factors, but this is controversial.
- The majority of the cardiac complications of ESRD are also clearly due to non-HD factors, such as diabetes, pre-existing hypertension, vascular disease – all of which lead to ESRD in the first place. Addressing HD session stability does little to affect these underlying processes which contribute to the primary outcome.
- Furthermore, the alternative of avoiding significant fluid removal (the intervention is designed in part to slow fluid removal) is often inadequate fluid removal, which can contribute to volume overload, hypertension, heart failure, and adverse cardiac events as well.

Criterion 2: Potential for the study to improve health care and outcomes

Reviewer 1:

Strengths:

-The application addresses knowledge gaps regarding ways to improve fluid management and to subsequently curtail session instability.

-The application also addresses the existing gap of knowledge regarding patients' roles in safety intervention.

-Partnership with the largest dialysis provider chain will facilitate timely dissemination and implementation of results.

-The intervention and implementation plan has been informed by preliminary studies by the

investigators.

Weaknesses:

-The discussion regarding variability addresses variability in outcomes and does not address variability in practice patterns.

Reviewer 2:

Strengths:

-The proposal focuses on the number of people who live with Chronic Kidney Disease (CKD), and who require dialysis, creating a large health care problem that is a burden on the patient in time, costs, and quality of life. The current number of people with CKD, which is the 9th leading cause of death in the US, is expected to continue to increase in a significant number. End Stage Renal Disease (ESRD), is a result of CKD , and often found as a comorbidity with hypertension and heart disease.

-Cardiovascular disease (CD) is a major cause of death among people with CKD and the proposal seeks ways to positively impact this percentage. The strain on the cardiovascular system to undergo hemodialysis (HD) poses complications which might be mitigated through the processes proposed for this funding request.

-Hemodialysis (HD) is used to treat patients with CKD but often with varying results that occur due to inconsistent treatment methods. The amount of time required of the patient to adhere to a dialysis regimen is substantial and creates a burden on the person, due to the requirements of time and resources.

Weaknesses:

-None noted.

Reviewer 3:

Strengths:

-The proposal uses a cluster randomized controlled trial design to investigate patient safety interventions to prevent hemodialysis session instability.

-The impact of chronic kidney disease (CKD) is substantial to patients, their families, and the health care system as CKD patients often have comorbidities, require dialysis which may impact their quality of life.

-Patient safety team training and patient activation to improve cardiovascular safety are innovative interventions as this addresses a current gap in the evidence for outpatient dialysis treatment/sessions.

-Should the proposal yield positive results, the findings could be disseminated and implemented quickly as the key stakeholders and investigators have both local and national reach and have designed the their study infrastructure to partner and engage stakeholders-including patients-from

the onset and throughout the each phase of the study.

Weaknesses:

-No weaknesses noted.

Reviewer 4:

Strengths:

- Both the patient peer mentoring intervention and the provider safety intervention work to engage these groups in positive ways to improve health system care delivery using well-validated behavioral theories.
- The focus on cardiovascular events is important, since cardiovascular disease is the primary cause of morbidity and mortality, despite this being a renal disease.

Weaknesses:

- The degree to which helping patients meaningfully participate in CV safety-related decisions such as fluid removal targets, session length and frequency will affect the CV outcomes seems proportionally small compared to the overall event rates. The authors estimate 20% relative-risk reduction (RRR) with a 3-4% absolute-risk reduction (ARR), but this number is likely to regress to the mean.
- The novelty is somewhat questionable. Variations of these interventions have been endorsed. Large HD corporations are already doing some internal pragmatic trials around these same issues.

Criterion 3: Technical merit

Reviewer 1:

Strengths:

-The team consists of clinicians and scientists with expertise in nephrology, nursing, patient safety trials, epidemiology, behavioral/social sciences, health disparities and peer mentoring.

-The interventions are based on valid theoretical frameworks.

-Comparison interventions are realistically selected.

-The sample size is appropriate and handling of missing data has been appropriately addressed.

Weaknesses:

-After the patient has received one or the other form of intervention, his/her role in implementing change in treatment to affect session instability is not clear.

-Sample size and power analysis are based only on interdialytic hypotension, while session instability is defined by many other patient-determined outcomes/symptoms. IDH might not result in any patient-perceived symptoms and many of the symptoms might not be associated with IDH.

-The ‘patient safety involvement behavior’ has not been well described or quantified. The description seems to reflect only adherence to diet and dialysis regimen.

Reviewer 2:

Reviewer 3:

Reviewer 4:

Strengths:

- The cluster-randomized clinical trial (CTRT) is an ideal design for HD interventions affecting dialysis centers. Patient-centered outcomes are already collected in routine care. HD is an excellent example of testing systems of healthcare delivery to improve patient outcomes. This example can then be tried in other more challenging settings.
- Multiple well-validated frameworks for peer mentoring and patient engagement are employed, including socio-cognitive theory and motivational interviewing. Similarly, the provider intervention is grounded in the theory of planned behavior. Implementation follows the knowledge to action framework.
- This project follows off the AHRQ-NOTICE project on HD infection control issues, which blazes the trail for testing a non-infection intervention in this setting.
- The combination of a nephrologist and a behavioralist has the potential to create good synergy. Both co-PI’s have a good record of success.

Weaknesses:

- The power of this study to detect a difference in the primary endpoint is questionable. The authors estimate a 20% RRR of the intervention versus usual care, which in-and-of-itself is fairly optimistic and based on small pilot data (see above comments about link between HD sessions and CV outcomes). This study involves 2x2 randomization of sites with a 3-month pre-period and 12-month post-period. However, the main hypothesis is that one intervention will be superior to the other, for which the study is not powered; the difference between two interventions designed to help optimize the same factors around HD session fluid removal, length and frequency will be much smaller than 20%.
- Diffusion / contamination of sites prior to randomization to the interventions sounds like a significant threat since these interventions appear to be partially accepted already at many sites. HD centers are not ignorant to the relevance of session stability; it is already a major focus of care efforts. This will further decrease power and raise chance of type 2 error.
- The investigators argue that the 2x2 factorial design is more efficient that the 3-arm trial. It seems that one advantage could be testing the synergy between the 2 interventions, which is not highlighted by the investigators.
- Peer mentoring has some overlap with the concept of patient navigators, which is explicitly not a current interest of PCORI. The peer-mentoring patient activation arm has multiple components, but it may help to provide additional detail as to how this arm is different than typical patient navigator interventions.
- The qualitative work in Aim 1 involves significant patient involvement, but is thinner on provider and center administrative involvement, despite the importance of both. The qualitative

research descriptions remain relatively superficial, even in this resubmission.

- The interventions do not offer an in-depth approach the multiple morbidity, mental health, substance related, and cognitive challenges faced by ESRD patients.
- The authors assume a >70% participation rate by patients at participating centers. This seems optimistic, particularly for an intervention like peer mentoring for which patients often decline.
- The application remains unclear about how it will handle patients who die or become ineligible for a variety of reasons in the analysis of non-mortality outcomes.
- The Potential Problems and Alternate Treatment section is relatively superficial for a 28-center >12-month intervention trial that involves engagement from patients, peer mentors, and complex provider teams. Center and provider engagement and retention promises to be a major challenge.
- This is really focused on HD session processes of care, without consideration of major alternative options to address ESRD. What about peritoneal dialysis (PD)? What about hospice? Granted, alternatives to HD are not the focus here; but what about 6x per week HD (somewhat supported in NEJM 2010 article)? Overall the question seems narrow and HD-center centric.

Criterion 4: Patient-centeredness

<u>Reviewer 1</u>:

Strengths:

-"Bad runs" and "crashes" have been identified by patients and providers as significant problems.

-Patients have expressed interest in ways to minimize 'crashes.'

Weaknesses:

-None noted.

<u>Reviewer 2</u>:

Strengths:

-Central to the proposal is the process in which patients can be educated and supported about the necessity of adherence to dialysis and the correct protocol. A portion of the difficulty with HD instability occurs with patient habits and adherence to treatment plans, and addressing those problems should create a positive outcome for patients.

-Patients needing HD face a number of problems, which the proposal identifies. The proposal documents the incidence of cardiovascular events, fatigue, nausea and prolonged time spent in medical care creates hardships for patients and their caregivers, and if the study is successful, these may be better managed and patient quality of life could improve.

-Empowering the patient through behavioral change, rather than using forms of instruction that may prove less effective, permits the patient to be at the center of their own care regimen regarding diet, habits, and adherence to dialysis appointments.

Weaknesses:

-None noted.

Reviewer 3:

Strengths

-As the proposal outlines, patient advisors are members of the project Steering Committee and provided key perspectives on the outcomes of interest to patients, particularly the peer mentoring format, evaluation measures, and study participation.

-The application addresses the patient centered question of options, benefits, and harms by engaging patients in a peer mentoring framework to assist in asking targeted questions and making decisions, and educating providers in patient safety techniques to improve HD instability and provide safer care.

-The proposal shares through patient focus groups that it was recognized that the improving cardiovascular safety of HD patients is important and needs improvement "to help patients feel better" (p. 3).

Weaknesses:

-No weaknesses noted.

Reviewer 4:

Strengths:

- The quality, including stability, of HD sessions is important to patients and intimately related to their quality of life.
- The secondary outcomes represent a good combination of events important to ESRD patients.
- The interventions are modified in Aim 1 to be patient and provider centric.

Weaknesses:

- The primary outcome, session stability, is more of a surrogate than a true endpoint.

Criterion 5: Patient and stakeholder engagement

Reviewer 1:

Strengths:

-Patients and other stakeholders, including the NKF as a patient advocacy organization, have been involved in various stages of the study design and will continue to contribute to the implementation and monitoring of the study.

-Patients have participated in formulation of the question, comparisons that should be made, length and format of the peer mentoring.

-Patients will be involved in the conduct of the study through involvement in the Steering Committee,

Advisory Board and the Data Safety Monitoring Board.

-Patients will provide feedback on disseminating important study results.

Weaknesses:

-None noted.

<u>Reviewer 2</u>:

Strengths:

-The National Kidney Foundation (NKF) is a key partner in this proposal and the organization has the reach to engage the diverse population of CKD patients in efficient and meaningful ways. The structure of NKF includes a strong patient presence in the development of materials and programs that are meaningful to the patient population.

-The addition of patient members to the governance structure for this proposal demonstrates the research team's efforts to include people living with CKF in all phases of the study.

-Using the existing peer mentoring model of the NKF and educating those mentors on the content relative to this proposal allows patient engagement on a personal and relevant level. The mentors are viewed as trusted voices based on their own experiences as HD patients and may connect emotionally and intellectually in a way that doctor to patient and clinic staff to patient might not achieve.

-The proposal clearly reflects the research team reviewed and adjusted their plan to include the patient in all aspects of the project.

Weaknesses:

None noted.

<u>Reviewer 3</u>:

Strengths:

-The patient engagement plan includes a project structure in which patient advisors and stakeholders are engaged as partners from formulating the research question, defining the study design, implementation, and dissemination.

-In addition, the study will provide compensation to both stakeholders and patients/families for their time and effort.

-The application details letters of support for this study from key stakeholders, patient advocate agencies, and patients.

Weaknesses:

-While a very minor weakness, it would helpful to understand the committee/advisory group reporting structure and how the groups communicate with one another for the betterment of the study

and patients. For example, how does each facility-based Operations Committee learn from each other in an efficient manner so that issues and resolutions identified at one facility can be shared with another, as appropriate.

Reviewer 4:

Strengths:

- There is good engagement of patients, peer mentors, families, providers, and even centers in the project, particularly in Aim 1.
- Partnership with the National Kidney Foundation (NKF) and dialysis facilities appears strong.

Weaknesses:

- The investigator-developed ktalk online community sounded compelling, but accessing the ktalk.org site 7/10/15 showed that the last new news posted was 8/7/13 and the last newsletter was 12/2011. On the forum, there are zero posts. It does not seem to be a currently vibrant source of patient input for this project. What else designed to show patient-engagement is lacking?

Overall Comments

Reviewer 1:

The proposal addresses knowledge gaps in an important day to day problem (session instability) impacting patient morbidity, mortality and quality of life in a large population affected by a major chronic illness, chronic kidney disease. This problem has been identified by patients and other stakeholders as significant. Patient and stakeholder engagement is appropriate and well defined and the proposal is patient-centered. The team is well-positioned to conduct the proposed research. The main weaknesses are in the technical merit: components of session instability, other than interdialytic hypotension, are not well defined as quantitative outcome measures. It has been proposed that the main impact of the interventions will occur through change in ‘patient safety involvement behavior’ which has not been well described or quantified. It is not clear how this measure will be different from adherence to diet and dialysis treatment.

Reviewer 2:

Accessible, actionable and influential is a phrase used in this proposal and is a great summary of the potential for this study. This proposal suggests there are meaningful ways to activate patient education and engagement, as well as provide substantive guidance to clinicians and dialysis center staffs, so that all parties involved with Chronic Kidney Failure and hemodialysis are sufficiently informed in ways to improve outcomes as they relate to cardiovascular disease.

If the methods proposed are found to have a favorable outcome the impact on the chronic kidney disease community would be significant and quickly implemented through education of patients, clinicians and clinic staff.

The inclusion of the National Kidney Foundation as a key member of this research team gives access to the greater CKF patient population as well as clinicians and clinic staff. The research of NKF gives credibility to the results and if found to be positive, an efficient and effective way to

rapidly disseminate study results.

Reviewer 3:

The proposal aims to prevent hemodialysis session instability, particularly improving cardiovascular safety, by testing a patient safety team training module and patient activation model using a cluster randomized control trials. The proposal also will track secondary patient-centered clinical outcomes such as patient symptoms, quality of life, mortality, etc. The study design, implementation, and dissemination engages key stakeholders including patient advocacy groups and patients advisors by involving them in the project committee and decision-making infrastructure. The application is well written, details the comprehensive and innovative interventions, and evaluates the intervention using patient-centered outcome measures. The study team is experienced in both clinical content and data analytics. The application is very strong in patient engagement, patient centeredness, and improving health systems.

Reviewer 4:

In summary, among patients with end-stage renal disease (ESRD), the investigators propose to compare the effectiveness of a multimodal provider education intervention (team training, online videos and educational activities, sample policies, and a safety procedure checklist) against a peer-mentoring patient activation intervention. The patient activation intervention extends a national peer mentoring program offered by the National Kidney Foundation, a partner. These interventions have largely been pilot tested in the inpatient environment, and will be adapted in Aim 1 to the outpatient setting using focus groups and interviews. The proposal would then test the comparative effectiveness of these two approaches in a pragmatic cluster-randomized controlled trial (CRCT) of 28 HD centers in 2x2 factorial design, with 3-month pre-period and 12-month post-period. The primary outcome is dialysis session safety in 1,400 patients in several US regions (Aim 2), with secondary outcomes of patient symptoms, fluid adherence, dialysis adherence, quality of life, hospitalizations and mortality over 12 months (Aim 3).

Overall, figuring out how to provide high-quality HD is a very important issue. The 6000+ HD facilities that provide HD to the >400,000 Americans with ESRD is an ideal environment for conducting cluster-randomized trials to that aim to improve health system care delivery. This study tests both a patient- and a provider-based intervention to improve HD session safety. These interventions have been previously studied and are being thoughtfully modified in a patient- and provider-centric way to fit the outpatient HD setting. This may inform similar ambulatory clinic-based interventions in other settings for other diseases.

The biggest concern is that HD centers already spend a lot of energy trying to optimize session stability and safety. There is likely significant diffusion of both interventions (peer mentoring and provider safety check lists) into current usual care, which will reduce the effectiveness of the intervention. The study is not powered to test differences between the 2 interventions, even though this is the primary stated goal (it is powered only against usual care). The investigators make a strong case for the association between ESRD and cardiovascular outcomes, but the relative importance of HD session instability to the main patient-centered outcomes is weak. Details on both the intervention modification and CRCT conduct are lacking, as is a serious consideration of practical challenges of completing the proposed 28-center study. Finally this study is nearly $7 million in cost.

Does the application have acceptable risks and/or adequate protections for human subjects?

Reviewer 1: Yes

Reviewer 2: Yes

Reviewer 3: Yes

Reviewer 4: Yes

Pragmatic cluster-randomized trial design, which creates certain ethical issues around patient agreement to participate. The authors skirt around this to some extent. Fortunately, the interventions are relatively accepted and patients can decline peer mentoring.
